# Emergence of multidrug-resistant and virulent *Escherichia coli* with APEC‑associated traits in broiler chickens from Ismailia, Egypt

**DOI:** 10.1038/s41598-026-45788-4

**Published:** 2026-04-11

**Authors:** Reham M. ELTarabili, Marwa E. Abo Hashem, Mona A. Ahmed, Fatma M. Yousseff, Mona S. Abdallah, Nada Hussein Eidaroos

**Affiliations:** 1 Department of Bacteriology, Immunology, and Mycology, Faculty of Veterinary Medicine, Suez Canal University, Ismailia, 41522 Egypt; 2 Department of Bacteriology, Immunology, and Mycology, Agriculture Research Center (ARC), Ismailia branch, Animal health research institute, Ismailia, 41511 Egypt; 3 Department of Clinical Pathology, Agriculture Research Center (ARC), Ismailia branch, Animal Health Research Institute, Ismailia, 41511 Egypt; 4 Avian and Rabbit Medicine Department, Faculty of Veterinary Medicine, Suez Canal University, Ismailia, 41522 Egypt

**Keywords:** *E. coli*, Broiler chickens, Antimicrobial resistance, Colibacillosis, MDR, ESBL, Microbiology, Molecular biology

## Abstract

Colibacillosis in poultry is a significant disease that leads to substantial economic losses and poses a potential zoonotic threat through the emergence of antimicrobial‑resistant strains. This study aimed to investigate the prevalence, antimicrobial resistance patterns, phylogenetic distribution, and virulence and resistance gene profiles of *Escherichia coli* isolates from broiler chickens on two private farms in Ismailia Governorate, Egypt. During April–July 2024, cloacal swabs were collected from diseased broiler chickens showing diarrhea and respiratory symptoms. Post-mortem examination revealed lesions consistent with colibacillosis, including Perihepatitis, Pericarditis, Enteritis, Splenomegaly, and Airsacculitis. *E. coli* was isolated and identified using selective culture media, biochemical assays, and PCR verification methods targeting the *pho*A gene. Antimicrobial susceptibility was assessed by the disc diffusion method against nine different antibiotic classes. Phylogenetic typing and PCR-based detection of virulence (*omp*A, *iss*, *iut*A, *iro*N, *eae*A, *kps*MTII) and resistance genes (*bla*_TEM_, *bla*_CTX−M_, *tet*A, *aad*A1, *sul*1, *qnr*A, *bla*_VIM−1_, *bla*_IMP−1_) were performed on the strains, and the data were statistically analyzed. Fifty-seven (28.5%) isolates were identified as *E. coli*, and both the hemolysis test and the Congo red binding test were positive for all isolates. Phylogenetic analysis showed that most isolates belonged to groups B2 (59.6%), B1 (21.1%), and D (19.3%). Notably, all isolates were resistant to ampicillin and tetracycline, while 93% of the isolates were resistant to cefuroxime and ceftriaxone. Overall, 100% were multidrug-resistant (MDR) strains, with a high MAR index (≥ 0.56). The most predominant resistance genes were *bla*_TEM_ and *tet*A (100%), followed by *bla*_CTX−M_ (91.2%) and *aad*A1 (85.9%). The main virulence determinants were *omp*A (100%), *iss* (78.9%), and *iut*A (64.9%). We observed correlations between phenotypic resistance, genotypic resistance, and the presence of virulence genes. The high burden of multidrug‑resistant isolates *E.coli* harbouring multiple virulence and extended‑spectrum β‑lactamase genes in Egyptian broilers underscores a serious threat to poultry production and public health. These findings highlight the urgent need for rational antimicrobial use, strengthened farm biosecurity, and continuous molecular surveillance within a One Health framework.

**Supplementary Information:**

The online version contains supplementary material available at 10.1038/s41598-026-45788-4.

## Introduction

*Escherichia coli* (*E. coli*) is a major pathogen worldwide in poultry, causing colibacillosis, a disease associated with significant economic losses, impaired growth, and increased mortality^1,2^. *E. coli* can act as a primary pathogen or as a secondary invader following viral infections (infectious bronchitis virus, Newcastle disease virus, avian influenza virus), bacterial infections (*Mycoplasma* spp.), or immunosuppressive conditions and environmental stressors such as overcrowding and high levels of dust and ammonia^1,2^.

Interestingly, *E. coli* can colonize the gastrointestinal and respiratory tracts of chickens without causing obvious disease and may only migrate to extraintestinal sites under stress conditions (including production-related stress, immunosuppression, or concurrent infection), thereby becoming an opportunistic pathogen^3,4^. The bacteria invade through abraded tracheal and intestinal epithelium, reach the bloodstream, and disseminate to internal organs. The transmission route is through contaminated feed and water, and further spreads to other poultry via faecal-oral or aerosol routes^4,5^.

*E. coli* typically inhabits the gastrointestinal tract of humans and animals; some strains have pathogenic potential^6^. Pathogenic variants are classified into enteropathogenic *Escherichia coli* (IntPEC) and extraintestinal pathogenic *Escherichia coli* (ExPEC). ExPEC strains characterized by having two or more virulence determinants, including *pap*A, *pap*C, *kps*MTII, and *iut*A^7^. A notable subgroup of ExPEC is avian pathogenic *E. coli* (APEC), frequently associated with antimicrobial resistance^7^. Genes more commonly detected in APEC include *iss*,* tsh*,* iro*N, *omp*T, *iut*A, *iuc*D and *papC*^8^.

The emergence of antimicrobial resistance in commensal bacteria such as *E. coli* poses a significant public health challenge, as resistance patterns in animal *E. coli* frequently mirror those in humans and can transfer antibiotic resistance genes to other pathogens^9,10^. Numerous studies, particularly those from Egypt, indicate that bacterial resistance to antibiotics such as β-lactams, fluoroquinolones, tetracyclines, and aminoglycosides is on the rise. Enterobacteriaceae exhibit drug resistance mechanisms, including the production of β-lactamases, particularly extended-spectrum β-lactamases (ESBLs) and class C (AmpC) β-lactamases^11^. This resistance frequently leads to co-resistance with additional antibiotics, thereby complicating therapeutic interventions^12^. The dissemination of ESBL-producing bacteria is influenced by the excessive use of antibiotics in humans and animals, as well as in agricultural practices, with food-producing animals serving as significant reservoirs. The rise of antibiotic-resistant *E. coli* strains in poultry presents an increasing challenge for managing their dissemination through antibiotic use. Significant factors include inadequate food hygiene, misuse and overuse of antibiotics, and lack of biosecurity and hygiene practices^13,14^.

The presence of antibiotic resistance alongside virulence factors in *E. coli* may increase its pathogenicity and complicate therapeutic interventions. Examining the relationship between resistance patterns and virulence genes is essential, as their interplay may affect bacterial survival, infection severity, and treatment difficulties^15^. Therefore, this study aimed to determine the prevalence, phylogenetic distribution, antimicrobial resistance patterns, virulence and resistance gene profiles, and their interrelationships in *E. coli* isolated from broiler chickens in Egypt, within a One Health context.

## Materials and methods

### Ethics statement

This study was conducted in compliance with the ARRIVE guidelines. All protocols were conducted in accordance with relevant guidelines and regulations. The study was carried out in accordance with the ARRIVE guidelines, and the experiment received approval from the Animal Ethics Review Committee of Suez Canal University (AERC-SCU), Egypt, under No. SCU-VET-AERC-R-2025031.

### Sample collection &Clinical and post-mortem examinations

A total of 200 cloacal swabs were collected from broiler chickens (one per bird) exhibiting clinical signs suggestive of colibacillosis. Birds were selected from two commercial broiler farms in Ismailia Governorate (100 per farm), Egypt, between April and July 2024, each with a flock size of approximately 1,000–5,000 birds. The affected flocks were at different production stages, ranging from 13 to 35 days of age and had routine vaccination programs. Sampling was performed during outbreaks with high morbidity and mortality rates. Within each affected flock, ten symptomatic birds were selected using Systematic sampling of clinically affected birds, that is, birds exhibiting clear clinical signs were chosen at regular intervals across different houses or sections of the flock to minimize location bias. No clinically healthy birds were included in the study. Cloacal swabs were collected under aseptic conditions and placed into appropriate transport medium (e.g. buffered peptone water) in sterile tubes, transported on ice, and processed within 24 h. Cloacal swabs were collected from live diseased birds. Post-mortem examination was subsequently performed on birds that had recently died.

### Isolation and identification of *E. coli*

Swabs were inoculated into McConkey’s broth (Oxoid, Hampshire, UK) and incubated for 24 h at 37 °C. A loopful of broth culture was inoculated onto MacConkey agar and eosin methylene blue agar (Oxoid, Hampshire, UK). The suspected colonies were identified based on their colonial characteristics, microscopic examination via Gram staining, motility tests, haemolytic activity on blood agar, and biochemical reactions (oxidase, catalase, indole, lactose fermentation, methyl-red, citrate utilization, H2S, Voges-Proskauer, and urease tests) as delineated by Quinn et al.^16^.

### Virulence characterization of *E. coli*

#### Haemolytic activity

A loop of *E. coli* culture was inoculated on blood agar to determine its haemolytic activity and pathogenicity^16^.

#### Congo-red binding test

The pathogenicity and invasiveness of the recovered *E. coli* isolates was evaluated using the Congo-red binding assay as outlined in prior research^17^. The isolated samples were inoculated onto tryptic soy agar with 0.03% Congo-red dye (Difco, USA) and incubated for 24 h at 37 °C. After incubation, the colonies were examined for red (positive) or white (negative) colonies.

#### Antimicrobial susceptibility testing

Using the standardized Kirby-Bauer disk diffusion technique, Muller-Hinton agar (Oxoid, Hampshire, UK) and nine antimicrobial agents (AMA) from eight different antimicrobial classes (AMC), the antimicrobial susceptibility patterns of *E. coli* isolates were determined according to the Clinical and Laboratory Standards Institute (CLSI). Antimicrobial susceptibility was interpreted using CLSI M100 (2020) breakpoints for Enterobacterales. Human clinical breakpoints were applied due to the absence of poultry-specific interpretive criteria for several agents tested. Nine antimicrobial discs were utilized and the antibiotic panel was selected based on clinical relevance to poultry medicine, and local availability as ampicillin (AMP, 10 µg), amoxicillin/clavulanic acid (AMC, 30 µg), imipenem (IPM, 10 µg), trimethoprim/sulfamethoxazole (SXT, 30 µg), gentamycin (CN, 10 µg), levofloxacin (LEV, 5 µg), tetracycline (TE, 30 µg), ceftriaxone (CRO, 30 µg), and cefuroxime (CXM, 30 µg). Multidrug resistance (MDR) is characterized by an isolate’s resistance to at least one agent across three or more unrelated antimicrobial classes^19^. MAR = (number of antibiotics to which the isolate is resistant) / (total number of antibiotics tested = 9)^20^ .

#### Molecular confirmation, phylotyping, resistance genes, and virulence genes of the isolated *E. coli* strains

Using QIAamp DNA Mini kit from Qiagen, Germany, to extract DNA from the isolated *E. coli* colonies, following the manufacturer’s instructions. A single pair of oligonucleotide primers from Thermo Fisher Scientific in Massachusetts, USA, was employed in standard PCR methods to confirm the presence of *E. coli* by targeting the *pho*A gene. Furthermore, the confirmed *E. coli* strains underwent phylotyping by amplifying the *chu*A, *yja*A, and *tsp*E4.C2 genes, as described by Clermont et al.^21^.

All PCR reactions were conducted using the Emerald Amp GT PCR master mix (Takara, CA, USA) according to the manufacturer’s guidelines. Conventional PCR amplification assays were performed on the molecularly confirmed *E. coli* isolates to determine the presence of genes associated with resistance to aminoglycosides (*aad*A), sulfonamides (*sul*1), tetracycline (*tet*A), quinolones (*qnr*A), β-lactams (*bla*_CTX−M_, *bla*_TEM_), and carbapenems (*bla*_VIM1_, *bla*_IMP1_). Additionally, virulotyping was performed through the detection of six significant virulence genes, namely *iss*, *iro*N, *omp*A, eaeA, *iut*A, and *kps*MTII, utilizing PCR assays. The primer sequences used in all PCR procedures are detailed in Table 1. In all PCR procedures, no‑template control served as the negative control, while *E. coli* ATCC 25,922 was used as the general positive control for species‑level targets.

**Table 1:** Sequences of primers and the amplified PCR products of target genes utilized in the PCR assay.

Specificity/ Target gene	Primer Sequence (5`–3`)	Amplified product (bp)	References
*E. coli*			
*pho*A	F: CGATTCTGGAAATGGCAAAAG	720	^22^
	R: CGTGATCAGCGGTGACTATGAC		
**Phylotyping**			
*tsp*E4.C2	F: GAGTAATGTCGGGGCATTCA	152	^21^
	R: CGCGYCAACAAAGTATTRCG		
*yja*A	F: TGAAGTGTCAGGAGAYGCTG	211	
	R: ATGRAGAATGCGTTCCTCAAC		
*chu*A	F: GACGAACCAACGGTCAGGAT	279	
	R: TGCCGCCAGTACCAAAGACA		
**Antimicrobial resistance genes**			
*sul*1	F: CGGCGTGGGCTACCTGAACG	433	^23^
	R: GCCGATCGCGTGAAGTTCCG		
*aad*A	F: TATCAGAGGTAGTTGGCGTCAT	484	^24^
	R: GTTCCATAGCGTTAAGGTTTCATT		
*tet*A	F: GGTTCACTCGAACGACGTCA	576	
	R: CTGTCCGACAAGTTGCATGA		
*qnr*A	F: ATTTCTCACGCCAGGATTTG	516	^25^
	R: GATCGGCAAAGGTTAGGTCA		
*bla*_CTX−M_	F: ATGTGCAGYACCAGTAARGTKATGGC	593	^26^
	R: TGGGTRAARTARGTSACCAGAAYCAGCGG		
*bla*_TEM_	F: ATCAGCAATAAACCAGC	516	^27^
	R: CCCCGAAGAACGTTTTC		
*bla*_VIM1_	F: AGTGGTGAGTATCCGACAG	261	^28^
	R: ATGAAAGTGCGTGGAGAC		
*bla*_IMP1_	F: ACCGCAGCAGAGTCTTTGCC	587	
	R: ACAACCAGTTTTGCCTTACC		
**Virulence genes**			
*ompA*	F: AGCTATCGCGATTGCAGTG	919	^29^
	R: GGTGTTGCCAGTAACCGG		
*iroN*	F: ATCCTCTGGTCGCTAACTG	847	
	R: CTGCACTGGAAGAACTGTTCT		
*kps*MTII	F: CAGGTAGCGTCGAACTGTA	280	
	R: CATCCAGACGATAAGCATGAGCA		
*eaeA*	F: ATGCTTAGTGCTGGTTTAGG	248	^30^
	R: GCCTTCATCATTTCGCTTTC		
*iss*	F: ATGTTATTTTCTGCCGCTCTG	266	^31^
	R: CTATTGTGAGCAATATACCC		
*iut*A	F: GGCTGGACATGGGAACTGG	300	
	R: CGTCGGGAACGGGTAGAATCG		

### Statistical analysis

The Chi-square test was employed for categorical data to determine the differences in the prevalence of phenotypic antimicrobial resistance, antimicrobial resistance genes, and virulence genes. Furthermore, the relationship among the examined criteria was assessed utilizing spearman’s correlation test using binary variables (presence/absence of resistance or genes, susceptible vs. resistant for each antibiotic). Graphs were produced using R software version 4.3.3 (https://www.r-project.org/).

## Results

### Clinical signs and post-mortem examination

**Fig. 1:**
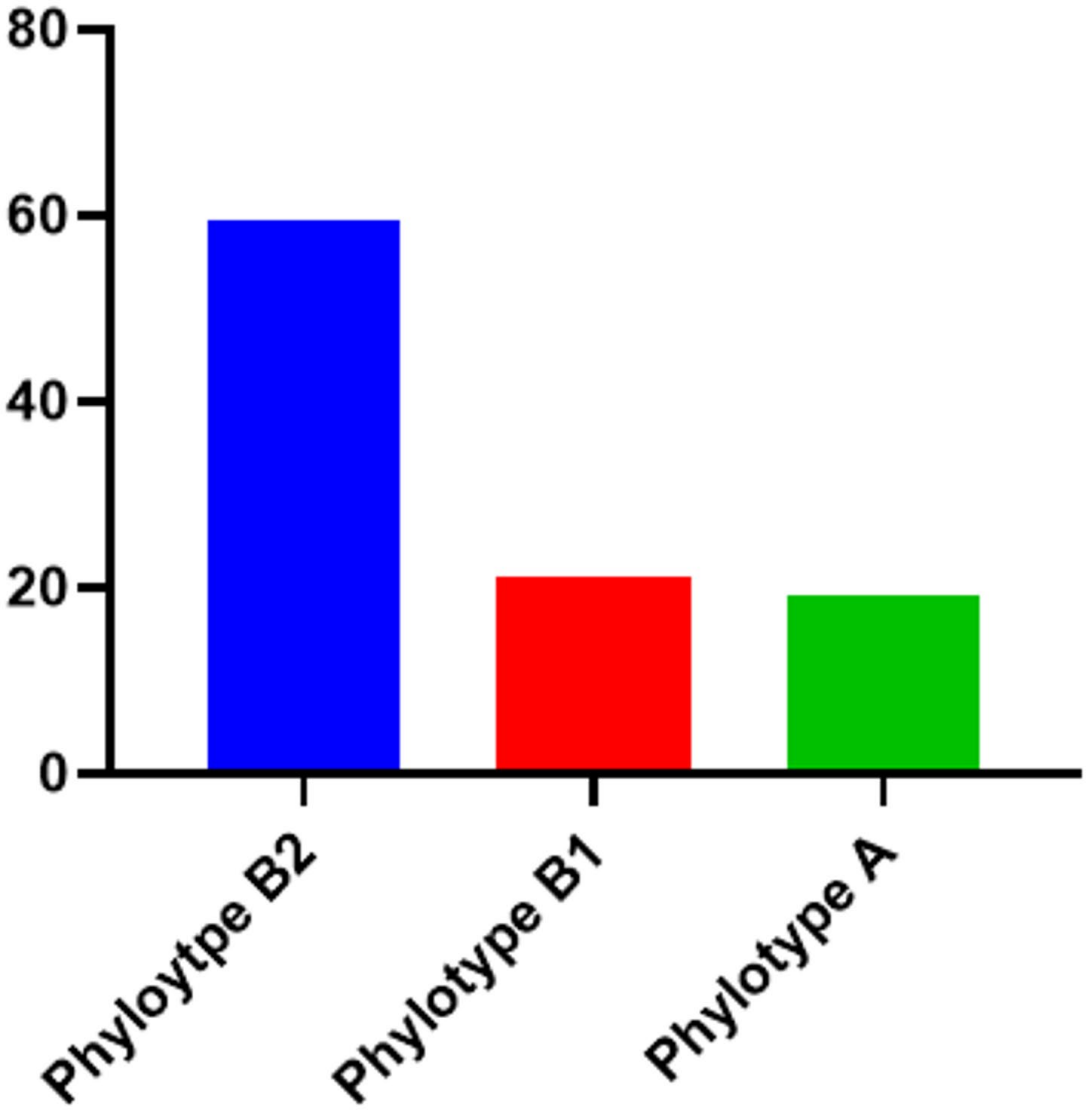
The figure displayed different *E. coli* phylotypes.

Diseased birds showed respiratory symptoms (gasping, coughing, sneezing, and rales), depression, dehydration, reduced feed intake, lethargy, ruffled feathers, drooping wings, reduced activity, decreased weight gain, and watery greenish diarrhoea. Post-mortem examination exhibited a classic sign of chronic Colibacillosis, including Perihepatitis (Fig. 1S), Pericarditis, Enteritis (Fig. 3S), Splenomegaly, indicating systemic infection (septicemia)(Fig. 4S), and Airsacculitis (Fig. 5S).

### Prevalence and bacterial characterization of *E. coli* isolates

The *E. coli* strains isolated in this study were all non-spore-forming, motile, and Gram-negative tiny bacilli. The colonies are relatively large and pink in colour on MacConkey agar, exhibit a green metallic sheen on eosin methylene blue agar, and are haemolytic on blood agar. In addition, all isolates tested positive for catalase, indole, methyl red, and nitrate reduction. Conversely, Oxidase, H2S, citrate utilization, urease, and VP assays were negative for all recovered isolates. A total of 200 cloacal samples from broilers were investigated. Of these, 57 isolates (28.5%) were confirmed to be *E. coli*.

### Phenotypic virulence characterization

All isolates exhibited virulence traits, including haemolysis on blood agar and a positive Congo red binding test.

### Phylotyping of *E. coli* isolates

All 57 *E. coli* isolates contained the *pho*A gene (a confirmed gene in the genus *E. coli*). Phylogenetic grouping analysis of 57 *E. coli* isolates showed that 34 isolates (59.6%), 12 isolates (21.1%), and 11 isolates (19.3%) belonged to phylogenetic groups B2, B1, and D, respectively (Fig. 1).

### Antimicrobial susceptibility profiles of *E. coli* isolates

**Fig. 2:**
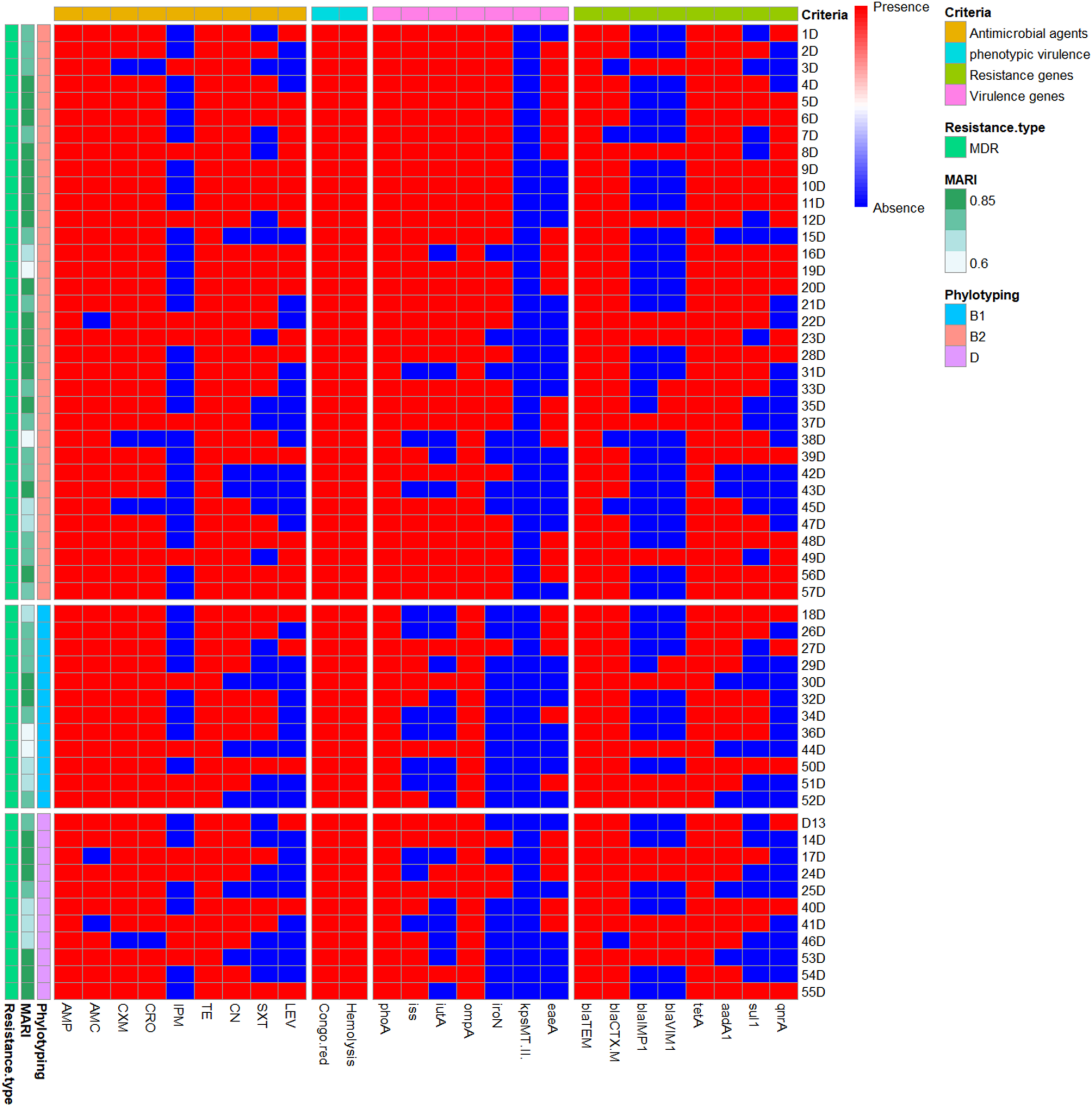
Heat map exhibiting distribution of 57 *E. coli* isolates of phylotyping, phenotypic, and genotypic resistance profiles and phenotypic and genotypic virulence profiles (intermediate resistance considered as sensitive). Red and blue colors represent the presence/absence of the phenotypic, genotypic resistance and virulence genes.

All *E. coli* isolates showed resistance to ampicillin and tetracycline (100% each), followed by resistance to amoxicillin/clavulanic acid (94.7%) and cefuroxime/ceftriaxone (93% each). Meanwhile, 56.1% of isolates were susceptible to levofloxacin and 72%of isolates were susceptible to imipenem (Tables 1 and 2S, and Fig. 2). Concerning various phylotypes of *E. coli* isolates, all *E. coli* isolates belonging to the B1 phylotype were resistant to amoxicillin and clavulanic acid, cefuroxime, and ceftriaxone (100% each). *E. coli* isolates belonging to phylotype B2 exhibited higher resistance rates than other phylotypes for levofloxacin, gentamycin, and Trimethoprim-sulfamethoxazole antimicrobials. Moreover, phylotype D isolates showed a higher frequency of imipenem resistance than other phylotypes. There were statistically significant variations in the resistance patterns between different phylotypes against all antimicrobials except imipenem (*p* < 0.001) (Table 2).

**Fig. 3:**
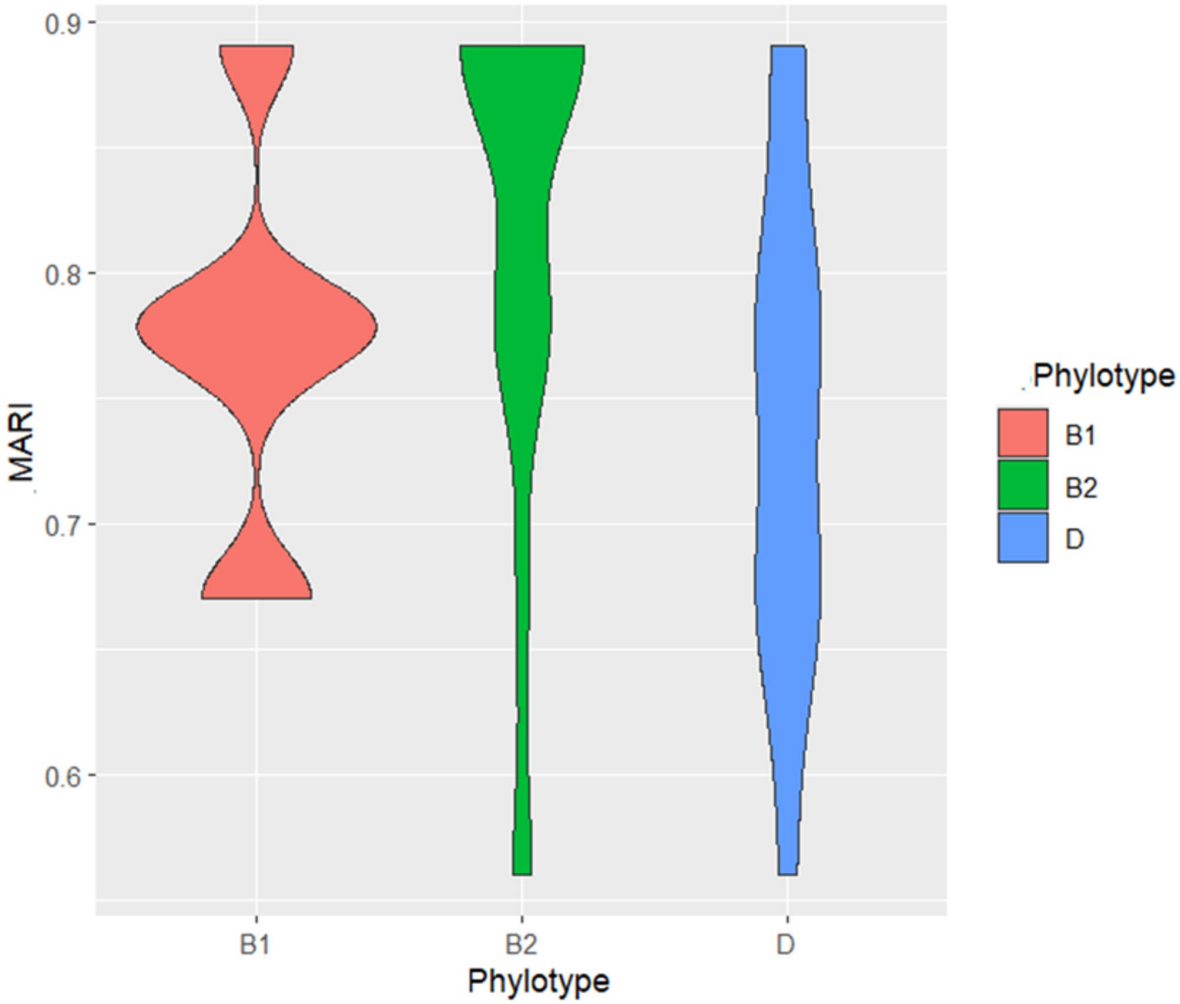
Multiple antibiotic resistance (MAR) indices of *E. coli* isolates.

In total, 100% of the tested *E. coli* isolates were MDR, with MAR indices ≥ 0.56 points, indicating high-risk contamination from antimicrobial utilization. Additionally, 36.8 and 42.1% of *E. coli* isolates were resistant to 8 and 7 antimicrobials with MAR indices of 0.89 and 0.78, respectively (Fig. 3; Table 3). The average MAR index of isolates phylotype B2 has the highest MAR index (0.82), followed by B1 (0.77) and D (0.74), with no significant differences (*p* > 0.05) (Fig. 3; Table 3).

Of note, the majority of *E. coli* isolates belonging to phylotypes B2 were resistant to 7 and 6 antimicrobial classes (50 and 35.3%, respectively), and B1 (16.7 and 58.3%, respectively) and D (18.2 and 36.4%, respectively) antimicrobial agents (Fig. 2). Additionally, 36.8 and 42.1% of *E. coli* isolates were resistant to 7 and 6 classes, respectively (Fig. 2; Table 3).

**Table 2:** Antimicrobial susceptibility of *E. coli*.

Antimicrobial class/ subclass	Antimicrobial agents	No. of resistant *E. coli* isolates (%)			*p*-value	Total no. of *E. coli* isolates (%) (*n* = 57)
		Phylotype B2 (*n* = 34)	Phylotype B1 (*n* = 12)	Phylotype D (*n* = 11)		
**Penicillins**	**Ampicillin**	34 (100)	12 (100)	11 (100)	*p* > 0.001	57 (100)
**β-lactams combination agents**	**Amoxicillin-clavulanic acid**	33 (97.1)	12 (100)	9 (81.8)	*p* > 0.001	54 (94.7)
**Cephalosporins ӀӀӀ**	**Ceftriaxone**	31 (91.2)	12 (100)	10 (90.9)	*p* > 0.001	53 (93)
	**Cefuroxime**	31 (91.2)	12 (100)	10 (90.9)	*p* > 0.001	53 (93)
**Carbapenems**	**Imipenem**	7 (20.6)	4 (33.3)	5 (45.5)	0.6456^NS^	16 (28)
**Fluoroquinolones**	**Levofloxacin**	19 (55.9)	3 (25)	3 (27.3)	*p* > 0.001	25 (43.9)
**Tetracyclines**	**Tetracycline**	34 (100)	12 (100)	11 (100)	*p* > 0.001	57 (100)
**Sulfonamides**	**Trimethoprim-sulfamethoxazole**	21 (61.8)	6 (50)	4 (36.4)	*p* > 0.001	31 (54.4)
**Aminoglycosides**	**Gentamycin**	31 (91.2)	9 (75)	9 (81.8)	*p* > 0.001	49 (86)

n= number, NS = Non significant, *p > 0.05,**p > 0.01,***p > 0.001.

**Table 3:** Multiple-antibiotic-resistance (MAR) indices of *E. coli* isolates belonging to various phylotypes.

MAR index (/9)	No. of resistant AMC	No. of resistant AMA	No. of resistant *E. coli* isolates (%)			Resistance pattern	Type of pattern	*p*-value	Total no. of *E. coli* isolates (%) (*n* = 57)
			Phylotype B2 (*n* = 34)	Phylotype B1 (*n* = 12)	Phylotype D (*n* = 11)				
5/9 0.56	4	5	2 (5.9%)	0	1 (9.1%)	AMP, AMC, CXM, CRO, and TE	MDR	0.3679^NS^	3 (5.3)
6/9 0.67	5	6	2 (5.9%)	0	3 (27.3%)	AMP, AMC, CXM, CRO, CN, and TE	MDR	0.2466^NS^	5 (8.8)
6/9 0.67	5	6	0	3 (25%)	1 (9.1%)	AMP, AMC, CXM, CRO, IPM, and TE	MDR	0.1738^NS^	4 (7)
7/9 0.78	6	7	3 (8.9%)	2 (16.7%)	0	AMP, AMC, CXM, CRO, CN, LEV, and TE	MDR	0.2466^NS^	5 (8.8)
7/9 0.78	6	7	7 (20.6%)	4 (33.3%)	0	AMP, AMC, CXM, CRO, CN, SXT, and TE	MDR	*p* > 0.05	11 (19.3)
7/9 0.78	6	7	2 (5.9%)	1 (2.9%)	2 (18.2%)	AMP, AMC, CXM, CRO, CN, IPM, and TE	MDR	0.8187^NS^	5 (8.8)
7/9 0.78	6	7	1(2.9%)	0	2 (18.2%)	AMP, CXM, CRO, CN, IPM, SXT, and TE	MDR	0.3679^NS^	3 (5.3)
8/9 0.89	7	8	13 (38.2)	2 (16.7)	2 (18.2)	AMP, AMC, CXM, CRO, CN, LEV, SXT, and TE	MDR	*p* > 0.001	17 (29.8)
8/9 0.89	7	8	4 (11.8)	0	0	AMP, AMC, CXM, CRO, CN, SXT, IPM, and TE	MDR	*p* > 0.01	4 (7)

MAR: multiple antibiotic resistance; AMA: number of resistant antimicrobial agent. AMC: number of resistant antimicrobial class, *p > 0.05,**p > 0.01,***p > 0.001,NS: Non Significant.

### Virulotyping of *E. coli* isolates with APEC‑ like associated virulence traits

Among tested *E. coli* isolates, *omp*A gene was the most prevalent one (100%), followed by *iss* (78.9%), *iut*A (64.9%), *iro*N (54.4%), and *eae*A (47.4%) (Fig. 2), with no detection *kps*MT (II). All the examined *E. coli* isolates (100%) also harbored at least two virulence genes. The tested virulence genes were more abundant among *E. coli* isolates belonging to phylotype B2 than those belonging to other phylotypes. Additionally, 8 virulence gene profiles were determined between the examined *E. coli* isolates. 43.8% exhibited the most common virulence gene profile ( *pho*A, *iss*,* iut*A, *omp*A, and *iro*N) (Table 4). There is no statistically significant variation (*p* < 0.05) among virulence gene profiles of the *E. coli* phylotypes except the first one (Table 4).

**Table 4:** Virulence genes’ profiles among *E. coli* phylotypes.

Virulence gene profiles	No. of *E. coli* isolates (%)			*p*-value	Total no. of *E. coli* isolates (%) (*n* = 57)
	Phylotype B2 (*n* = 34)	Phylotype B1 (*n* = 12)	Phylotype D (*n* = 11)		
*pho*A, *iss*,* iut*A, *omp*A, *iro*N, and *eae*A	15 (44.1%)	1 (8.3%)	1 (9.1%)	*p* > 0.000	17 (29.8%5)
*pho*A, *iss*,* iut*A, *omp*A, and *iro*N	12 (35.3%)	7 (58.3%)	6 (54.5%)	0.2894^NS^	25 (43.8%)
*pho*A, *iut*A, *omp*A, *iro*N, and *eae*A	0	0	1 (9.1%)	0.3679^NS^	1 (1.8%)
*pho*A, *iss*,* omp*A, and *iut*A	2 (5.9%)	0	0	0.1353^NS^	2 (3.5%)
*pho*A, *iss*,* omp*A, and *eae*A	1 (2.9%)	0	1 (9.1%)	0.1353^NS^	2 (3.5%)
*pho*A, *omp*A, and *eae*A	1 (2.9%)	4 (33.3%)	2 (18.2%)	0.3679^NS^	6 (10.5%)
*pho*A, *iss*, and *omp*A	1 (2.9%)	0	0	0.3679^NS^	1 (1.8%)
*pho*A, and *omp*A	2 (5.9%)	0	0	0.1353^NS^	2 (3.5%)

n= number.,*p > 0.05,**p > 0.01,***p > 0.001,NS : Non Significant.

### Detections of antimicrobial resistance of APEC isolates

Among tested *E. coli* isolates, *bla*_TEM_ and *tet*A genes were the most prevalent (100%), followed by *bla*_CTX−M_ (91.2%), *aad*A1 (86%), *sul*1 (54.4%), *qnr*A (43.9%), *bla*_VIM−1_(33.3%), and *bla*_IMP−1_ (28%) (Fig. 2). All the examined *E. coli* isolates (100%) also harboured at least three resistance genes. The tested resistance genes were more abundant among *E. coli* isolates belonging to phylotype B2 than those belonging to other phylotypes. Additionally, 14 resistance genes’ profiles were determined between the examined *E. coli* isolates. 26.3% exhibited the most common resistance gene profile (*bla*_TEM_, *bla*_CTX−M_, *tet*A, *aad*A1, *qnr*A, and *sul*1 ) (Table 5). There is no statistically significant variation (*p* < 0.05) among resistance gene profiles of the *E. coli* phylotypes except for two profiles (Table 5).

**Table 5:** Resistance genes’ profiles among *E. coli* phylotypes.

Resistance gene profiles	No. of *E. coli* isolates (%)			*p*-value	Total no. of *E. coli* isolates (%) (*n* = 57)
	Phylotype B2 (*n* = 34)	Phylotype B1 (*n* = 12)	Phylotype D (*n* = 11)		
*bla*_TEM_, *bla*_CTX−M_, *bla*_IMP1_, *bla*_VIM1,_ *tet*A, *aad*A1,and *qnr*A	3 (8.8%)	0	1 (9.1%)	0.1738^NS^	4 (7.1%)
*bla*_TEM_, *bla*_CTX−M_, *bla*_IMP1_, *bla*_VIM1,_ *tet*A, *aad*A1, and *sul*1	2 (5.9%)	1 (8.3%)	0	0.3679^NS^	3 (5.3%)
*bla*_TEM_, *bla*_CTX−M_, *tet*A, *aad*A1, *qnr*A and *sul*1	10 (29.4%)	2 (16.7%)	5 (45.4%)	0.056	15 (26.3%)
*bla*_TEM_, *bla*_CTX−M_, *bla*_VIM1,_ *tet*A, and *aad*A1	1 (2.9%)	1 (8.3%)	1 (9.1%)	-	3 (5.3%)
*bla*_TEM_, *bla*_CTX−M_, *tet*A, *aad*A1, and *sul*1	8 (23.5%)	1 (8.3%)	1 (9.1%)	0.007447	10 (17.5%)
*bla*_TEM_, *bla*_CTX−M_, *bla*_IMP1_, *bla*_VIM1,_ and *tet*A	1 (2.9%)	1 (8.3%)	2 (18.2%)	0.7788^NS^	4 (7.1%)
*bla*_TEM_, *bla*_IMP1_, *bla*_VIM1,_ *tet*A, and *aad*A1	1 (2.9%)	1 (8.3%)	0	0.1353^NS^	2 (3.5%)
*bla*_TEM_, *bla*_CTX−M_, *bla*_VIM1,_ *tet*A, and *aad*A1	1 (2.9%)	1 (8.3%)	0	0.1353^NS^	2 (3.5%)
*bla*_TEM_, *bla*_CTX−M_, *tet*A, *aad*A1, and *qnr*A	3 (8.8%)	0	0	0.1738^NS^	3 (5.3%)
*bla*_TEM_, *tet*A, *aad*A1, and *qnr*A	1 (2.9%)	0	0	0.3679^NS^	1 (1.8%)
*bla*_TEM_, *bla*_CTX−M_, *tet*A, and *aad*A1	1 (2.9%)	0	1 (9.1%)	0.1353^NS^	2 (3.5%)
*bla*_TEM_, *tet*A, *sul*1 and *aad*A1	0	1 (8.3%)	0	0.3679^NS^	1 (1.8%)
*bla*_TEM_, *bla*_CTX−M_, and *tet*A	2 (5.9%)	2 (16.7%)	0	0.3679^NS^	4 (7.1%)
*bla*_TEM_, *tet*A, and *aad*A1	0	1 (8.3%)	0	0.3679^NS^	1 (1.8%)

n= number.,*p > 0.05,**p > 0.01,***p > 0.001,NS : Non Significant.

### Correlation analysis and clustering of antimicrobial resistance phenotypes, antimicrobial resistance genes, and virulence genes among different phylotypes

**Fig. 4:**
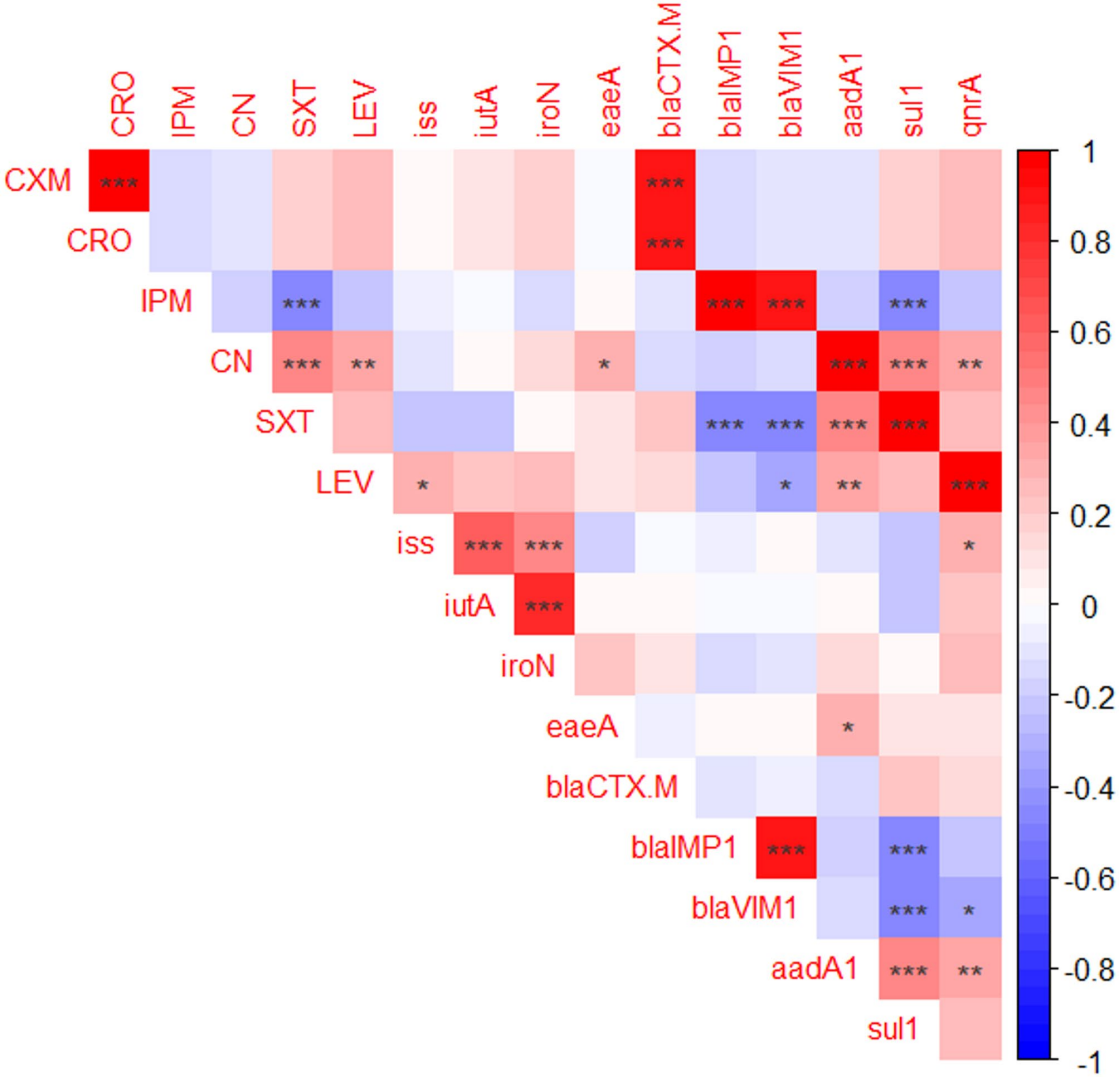
The correlation among virulence genes of *E. coli*, the antimicrobial resistance gene, phenotypes of antimicrobial resistance, and resistance patterns. Blue indicates a negative association while red indicates a positive one. ). The darker red and blue colors indicate stronger positive and negative correlations. The significance of the correlation is indicated by the stars: *p < 0.05, **p < 0.01, ***p < 0.001.

The figure shows the correlation analysis among different phylotypes. Resistance to ampicillin, tetracycline, Congo red, hemolysis, and prevalence of *omp*A, *pho*A, *bla*_TEM_ and *tet*A genes were excluded from the correlation analysis because they were identical across all isolates. The most significant correlations among the antimicrobials were CRO and CXM (*r* = 1), CN and SXT (*r* = 0.44), and CN and LEV (*r* = 0.36). The strongest positive associations between virulence genes were *iut*A and *iro*N (*r* = 0.80), *iss* and *iut*A (*r* = 0.61), and *iroN* and *iss* (*r* = 0.48). The strongest positive associations between resistance genes were *bla*_IMP−1_ and *bla*_VIM−1_ (*r* = 1), *aad*A1 and *sul*1(*r* = 0.44), and *aad*A1 and *qnr*A (*r* = 0.36) (Fig. 4).

**Fig. 5:**
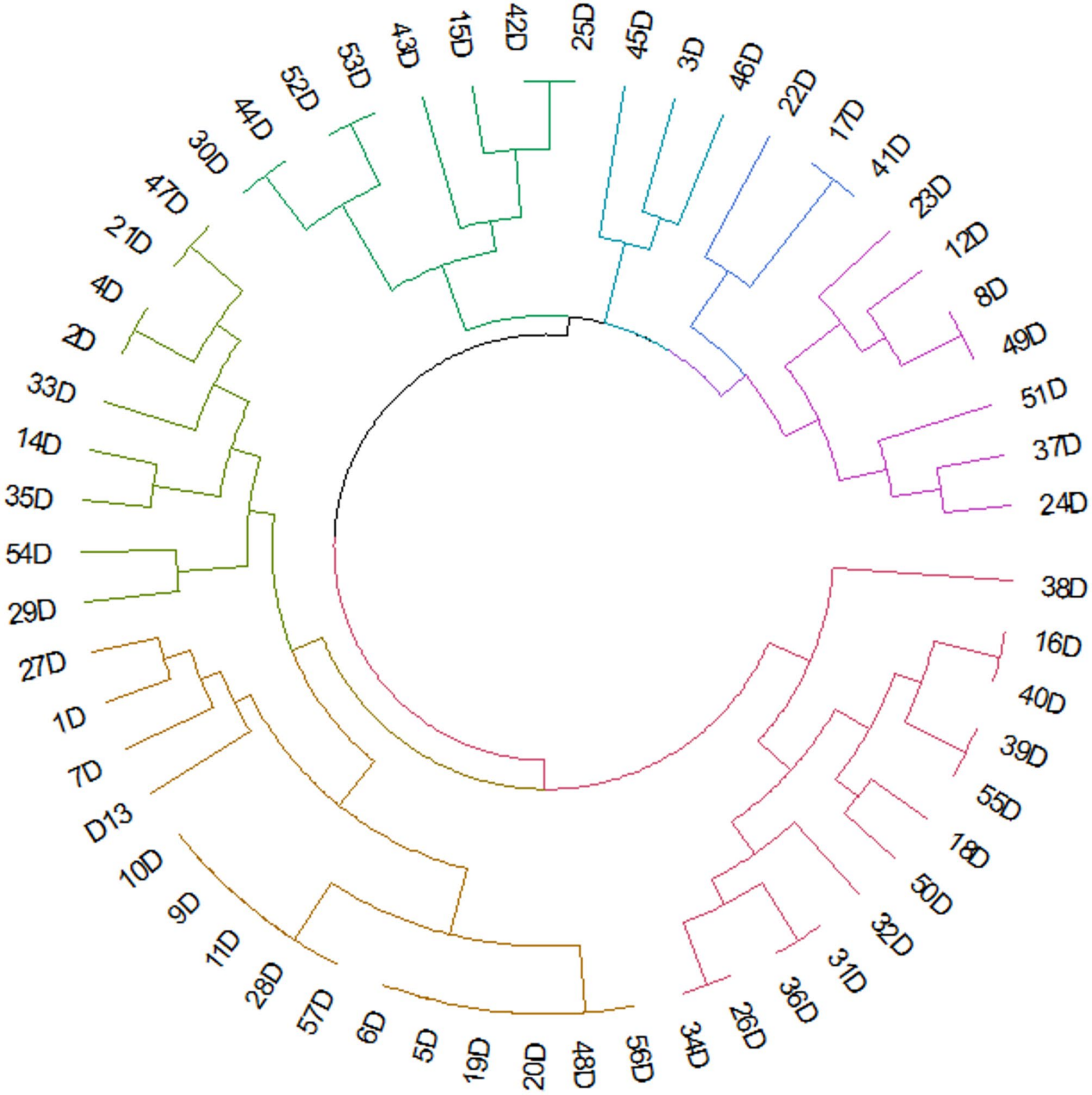
Circular dendogram exhibiting hierarchrical clustering of 57 *E. coli* phylotypes among the distribution of antimicrobial resistance (AMR) phenotypes, genotypes, virulence phenotypes, and genes.

Positive correlations were found between phenotypic and genotypic resistance, particularly between SXT and *sul*1(*r* = 1), LEV and *qnr*A(*r* = 1), CN and *aad*A1(*r* = 1), IPM and *bla*_IMP_ (*r* = 1), CRO, CXM and *bla*_CTX−M_ (*r* = 0.89), IPM and *bla*_VIM_ (*r* = 0.88). There are significant correlations between virulence genes and phenotypic resistance: CN and *eae*A(*r* = 0.28). There are no significant correlations between virulence genes and genotypic resistance (Fig. 4). The *E. coli* phylotypes investigated clustered into seven groups based on antimicrobial resistance (AMR) phenotypes, genotypes, virulence phenotypes, and genes (Fig. 5).

## Discussion

*E. coli* causes colibacillosis in poultry, resulting in economic losses and zoonotic hazards due to drug-resistant strains. This study reveals a concerning epidemiological profile of *E. coli* in broiler farms, characterized by high prevalence (28.5%), extensive antimicrobial resistance, and significant virulence potential. The 28.5% isolation rate of *E. coli* from clinically affected broilers aligns with previous Egyptian reports^32,33^, suggesting endemic circulation of *E. coli* in commercial flocks. All isolates exhibit hemolytic activity and Congo red positive, which function as phenotypic indicators of pathogenicity, aligning with the invasive potential outlined by Berkhoff & Vinal^34^ and El-Tarabili et al.^35^. Congo red specifically indicates the presence of virulence-associated surface features that promote bacterial adhesion and invasion, which are essential initial steps in the pathogenesis of colibacillosis. The consistency of these phenotypic markers across all isolates indicates selection for particularly virulent clones within Egyptian agricultural systems, potentially influenced by intensive farming conditions that promote pathogen transmission.

The phylogenetic composition of our isolates reveals a high predominance of group B2 (59.6%), followed by B1 (21.1%) and D (19.3%). Only B1, B2, and D were detected in our isolates, and the absence of A and E may reflect our limited sample size and sampling of diseased birds. This distribution differs significantly from that observed in European studies, where phylogroups A and B1 generally predominate in poultry^36^. The high prevalence of B2 and D phylogroups is significant because these groups are commonly associated with extraintestinal infections in humans and are known to harbour extensive virulence arsenals^7,29^. Recent investigations support this pattern: Wang et al.^37^ reported B2 as the dominant phylogroup (64.8%) among uropathogenic *E. coli*, with strains carrying multiple virulence factors. Similarly, Luo et al.^38^ found that phylogroup B2 isolates harboured more than three virulence genes, followed by group D with two to three factors.

The antimicrobial resistance patterns observed in this study are alarming from both veterinary and public health perspectives. High resistance to ampicillin (100%) and tetracycline (100%), coupled with extremely high resistance to third-generation cephalosporins (ceftriaxone and cefuroxime, 93%) and β-lactam/β-lactamase inhibitor combinations (amoxicillin-clavulanic acid, 94.7%). In contrast, resistance to carbapenems (imipenem, 28%) and fluoroquinolones (levofloxacin, 43.9%) was relatively low. Comparable resistance trends have been reported. For instance, Kocúreková et al.^36^ observed dominant resistance to tetracycline (49%), ampicillin (66%), and ciprofloxacin (61%) in avian isolates, along with sporadic resistance to cephalosporins, aminoglycosides, and colistin. Likewise, Sorour et al.^39^ documented MDR among *E. coli* isolates, with particularly high resistance to penicillins, cephalosporins, doxycycline, and gentamycin. Ahmed et al.^40^ also reported that Egyptian chicken isolates exhibited high resistance to ampicillin, amoxicillin–clavulanic acid, chloramphenicol, and ceftazidime, with > 94% resistance to tetracycline, trimethoprim–sulfamethoxazole, and cefotaxime. Moderate resistance was observed to doxycycline, nalidixic acid, and ciprofloxacin, whereas colistin and gentamycin showed lower resistance. Several studies documented high resistance to β-lactams, particularly ampicillin,^41,42^, while Abdel-Rahman et al.^43^ and Halfaoui et al.^44^. also identified moderate resistance to cephalosporins in North African poultry isolates. However, discrepancies when compared with other studies reflect differences in geographic location, antimicrobial stewardship policies, and the extent of antibiotic misuse in animal husbandry. For example, Irenge et al.^45^ reported only 1% resistance to amoxicillin–clavulanic acid and 65% resistance to ceftazidime in Congolese isolates, while Falgenhauer et al.^46^ found resistance levels of 56.8% for ceftriaxone and 54.5% for ciprofloxacin in cattle isolates from USA.

The phylogroup-specific resistance profiles provide mechanistic insights: phylogroup B1 exhibited universal resistance (100%) to amoxicillin-clavulanic acid, cefuroxime, and ceftriaxone, while B2 isolates demonstrated higher resistance to levofloxacin, gentamycin, and trimethoprim-sulfamethoxazole. This phylogenetic stratification of resistance suggests distinct evolutionary trajectories and selective pressures operating within each lineage. The finding that phylogroup D isolates showed elevated carbapenem resistance (imipenem) is particularly concerning, as carbapenems represent last-resort antibiotics for treating serious Gram-negative infections. The average MAR indices as B2 (0.82), B1 (0.77), and D (0.74), which exceed the 0.2 threshold, indicating high-risk contamination sources with intensive antibiotic exposure^47^.

The species-specific gene, *pho*A, serves as the housekeeping gene for *E. coli* and is used to confirm the *E. coli* strains. The *pho*A target gene has been established as the universal marker for *E. coli*, corroborated by multiple studies with no detection in non-*E. coli* strains^48^. In *E. coli*, the *pho*A gene has been widely used as a molecular marker in studies of protein localization and membrane topology, as its enzymatic product is active only when exported from the plasma membrane^49^. Rathi et al.^50^; Thong et al.^51^;Saeed et al.^52^ confirm its specificity and accuracy in distinguishing *E. coli* from other enteric bacteria. Previous studies of Hu et al.^53^; Alnahass et al.^54^; Afayibo et al.^55^ employed this gene to identify pathogenic *E. coli* in samples obtained from both healthy and diseased chickens. In the present study, all 57 *E. coli* isolates were found to harbor the *pho*A gene, confirming their identity as *E. coli*, supporting the reliability and specificity of this marker for species confirmation.

The virulence gene profiles identified in our study reinforce this phylogenetic-pathogenic correlation. The ubiquitous *omp*A gene (100%) encodes outer membrane protein A, which mediates adhesion, invasion, and serum resistance as fundamental virulence mechanisms in systemic infections. The high prevalence of *iss* (78.9%), which encodes the increased serum survival protein, and *iut*A (64.9%), involved in aerobactin-mediated iron acquisition, further characterizes these isolates as possessing typical APEC virulence attributes. The *eae*A gene, which encodes the adhesin intimin, is associated with attaching and effacing (A/E) lesions in the intestinal tract of chickens infected with Avian Pathogenic *E. coli* (APEC), which is part of the “locus of enterocyte effacement” (LEE) pathogenicity island, as described in a previous study by Kiliç et al.^56^.The predominant virulence profile (*pho*A, *iss*, *iut*A, *omp*A, *iro*N, and *eae*A; 29.8%) represents a particularly concerning combination, as these genes synergistically enhance bacterial survival in host tissues, immune evasion, and colonization capacity.

In the present study, the most frequently detected resistance genes in *E. coli* isolates were *bla*_TEM_ and *tet*A (100%), followed by *bla*_CTX−M_ (91.2%), *aad*A1 (85.9%), *sul*1 (54.4%), *qnr*A (43.9%), *bla*_VIM−1_ (33.3%), and *bla*_IMP−1_ (28%). Among extended-spectrum β-lactamase (ESBL) genes, *bla*_CTX−M_ and *bla*_TEM_ (83.3%) were the most dominant. These findings are consistent with earlier studies. In Japan, CTX-M- and TEM-type ESBLs were widely identified in both livestock and companion animals^28^. Egyptian reports have also confirmed the presence of *bla*_TEM_ in *Enterobacteriaceae* from healthy poultry^57^, while poultry offal was found to harbour ESBL-producing *E. coli* carrying *bla*_CTX−M_^58^. Furthermore, ESBL producers are often co-resistant to quinolones, aminoglycosides, tetracyclines, chloramphenicol, and trimethoprim, mainly due to the dissemination of mobile genetic elements such as plasmids, integrons, and transposons^59^. Previous studies reported *bla*_CTX−M_ in 72.5% of community-acquired isolates^60^. In Spain, Valverde et al.^61^ reported that 70% of healthy individuals were colonized with ESBL-producing *Enterobacteriaceae* harboring *bla*_CTX−M_, while Ahmed et al.^62^ showed its high prevalence (96.6%) among community isolates in Libya.

The high prevalence of aminoglycoside *(aad*A1, 85.9%) and sulfonamide (*sul*1, 54.4%) resistance genes reflects the historical and ongoing use of these antimicrobial classes in Egyptian poultry production. The detection of plasmid-mediated quinolone resistance genes *(qnr*A, 43.9%) is noteworthy, as this mechanism enables low-level fluoroquinolone resistance, potentially leading to high-level resistance via chromosomal changes. The detection of carbapenemase genes (*bla*_VIM−1_, 33.3% and *bla*_IMP−1_, 28%) in poultry-derived *E. coli* constitutes a significant public health risk, as carbapenem resistance drastically restricts treatment options for human infections. The discrepancy between phenotypic resistance to imipenem (28%) and carbapenemase gene (*bla*_VIM−1_) carriage (33.3%) may be due to some *bla*_VIM_‑positive isolates that may still test ‘susceptible’ or only intermediate in routine disk assays if enzyme expression is low and alternative resistance mechanisms.

Our study revealed 100% of the tested *E. coli* isolates were classified as MDR with MAR indices ≥ 0.56, indicating high-risk contamination sources linked to antimicrobial usage. Resistance to eight and seven antimicrobials was detected in 36.8% and 42.1% of isolates, respectively, with corresponding MAR indices of 0.89 and 0.78. The average MAR index was highest in phylotype B2 (0.82), followed by B1 (0.77) and D (0.74). Notably, most *E. coli* isolates of phylotype B2 showed resistance to 7 (50%) and 6 (35.3%) antimicrobial classes, respectively. In comparison, phylotype B1 exhibited resistance to seven (16.7%) and six (58.3%) classes, while phylotype D showed resistance to seven (18.2%) and six (36.4%) classes. Agusi et al.^63^ reported a high level of MDR, which was largely attributed to the misuse and overuse of these antibiotics in animal production. Conversely, lower resistance rates to ceftriaxone, cefotaxime, chloramphenicol, ciprofloxacin, and gentamycin were observed, possibly reflecting their higher cost and more restricted routes of administration. In the present study, tetracycline resistance was notably prevalent among *E. coli* isolates, a finding that aligns with Amer et al.^32^. The widespread resistance to tetracycline is likely linked to its extensive and prolonged use as a first-line therapeutic and prophylactic antibiotic. Furthermore, the frequent detection of the *tet*A gene in MDR *E. coli* isolates in this study corroborates earlier findings by Algammal et al.^17^ in livestock, underscoring the role of genetic determinants in mediating resistance dissemination.

The correlation analysis revealed strong associations among virulence determinants, particularly between *iut*A and *iro*N (*r* = 0.80) and between *iss* and *iut*A (*r* = 0.61), suggesting that co-selection of iron-acquisition and serum-resistance factors is critical for systemic pathogenesis. Notably, these virulence traits showed positive correlations with XDR phenotypes (*iro*N *r* = 0.43, *iut*A *r* = 0.39, *iss* *r* = 0.33), indicating that the most resistant isolates also possess the greatest pathogenic potential. This concerning convergence of resistance and virulence traits likely reflects co-localization of these determinants on mobile genetic elements such as plasmids and pathogenicity islands, facilitating their simultaneous dissemination within bacterial populations^59,64^.

While these correlations are strong, it should be noted that the limited sample size (*n* = 57) may contribute to some of the perfect correlations observed (*r* = 1.0), particularly trimethoprim-sulfamethoxazole with *sul*1 (*r* = 1.0), levofloxacin with *qnr*A (*r* = 1.0), gentamycin with *aad*A1 (*r* = 1.0), and cephalosporins with *bla*_CTX−M_ (*r* = 0.89), validate the predictive utility of resistance gene screening. In our isolates, *sul*1 was strongly associated with the SXT‑resistant phenotype, consistent with previous studies of Antunes et al.^65^; El-Tarabili et al.^66^ who found that *sul*1 is part of the conserved region of class 1 integrons. Although we did not directly test *dfr* genes, the tight genetic linkage between *sul*1 and *dfr*A in these elements means that *sul*1 can act as a practical marker of SXT resistance in this collection. Moreover, the perfect correlation between carbapenemase genes *bla*_IMP−1_ and *bla*_VIM−1_ (*r* = 1.0) suggests these determinants may^65^ be co-located on the same mobile genetic elements, facilitating their co-transmission. Correlation analysis showed strong associations between resistance genes and phenotypic resistance, such as ESBL genes (*bla*_CTX−M_, *bla*_TEM_, and *bla*_SHV_). These findings highlight the role of resistance and virulence traits, facilitating the emergence and spread of highly virulent multidrug-resistant *E. coli* strains^59,64^. The association between *aad*A1 and *sul*1 (*r* = 0.44) and between *aad*A1 and *qnr*A (*r* = 0.36) supports this integron-mediated co-resistance hypothesis.

The phylogenetic clustering analysis revealed seven distinct groups based on antimicrobial resistance phenotypes, genotypes, and virulence profiles, indicating complex evolutionary dynamics within the *E. coli* population, possibly reflecting exchange between environmental, commensal, and pathogenic *E. coli* populations. The identification of phylogroup B2 isolates carrying the most extensive resistance and virulence arsenals represents a worst-case scenario, combining enhanced pathogenic potential with treatment recalcitrance.

## Conclusion

This study demonstrates the widespread dissemination of MDR *E. coli* isolates in two broiler farms, predominantly belonging to phylogroups B2 and D and harboring extensive repertoires of ESBL and virulence genes. The detection of carbapenemase genes in poultry *E. coli* represents a critical breach of antimicrobial stewardship principles, as these last-resort drugs should be reserved exclusively for human medicine. These isolates exhibit high resistance to β-lactam, tetracycline, and cephalosporin antibiotics and widely carry extended-spectrum β-lactamase (ESBL) genes, such as *bla*_TEM_ and *bla*_CTX−M_. The predominance of virulence determinants (*omp*A, *iss*,* iut*A, *iro*N) within phylogroups B2 and D underscores their enhanced pathogenic potential. The strong correlations among phenotypic resistance, resistance genes, and virulence variables indicate co-selection and development of hazardous resistant clones. These findings underscore the necessity of prudent antibiotic application in poultry production, biosecurity measures, and genetic monitoring to avert the zoonotic transmission of resistant APEC strains into the food supply and environment. These findings support the growing literature indicating that antibiotic resistance constitutes a One Health challenge that necessitates a comprehensive approach to human, animal, and environmental health. Only comprehensive, multi-sectoral strategies can alter resistance, protecting both animal and human health in Egypt and worldwide.

### Limitations

The present study has several limitations that should be considered when interpreting the findings. First, sampling was restricted to cloacal swabs from clinically affected broilers on two private farms in Ismailia Governorate and may not be representative of all Egyptian poultry production systems. Second, *pho*A is specific; however, there is a need for additional markers (e.g., *uid*A/*usp*A/16S‑based *E. coli*‑specific targets or MALDI‑TOF) that would further strengthen confirmation. Third, Colistin, florfenicol, and enrofloxacin/ciprofloxacin are relevant in poultry but were not included due to resource limitations, and the absence of these agents restricts our ability to fully assess resistance. Fourth, our findings regarding *bla*_VIM‑1_ and *bla*_IMP‑1_ are based on conventional PCR only as preliminary and must be interpreted with sequencing or WGS in future studies with MICs to detect phenotypic carbapenemase assays. Finally, Virulence genes, including *pap*C, *hly*F, *omp*T, *iuc*D, and *tsh* were not evaluated. The absence of these data may have restricted the completeness of the ExPEC/APEC schemes.

## Supplementary Information

Below is the link to the electronic supplementary material.

Supplementary Material 1

## Data Availability

The data supporting this study’s findings are available from the corresponding author upon request.

## References

[1] Dho-Moulin, M. & Fairbrother, J. M. Avian pathogenic Escherichia coli (APEC). *Vet. Res.* **30**, 299–316 (1999). 10367360

[2] Ghunaim, H., Abu-Madi, M. A. & Kariyawasam, S. Advances in vaccination against avian pathogenic Escherichia coli respiratory disease: potentials and limitations. *Vet. Microbiol.* **172**, 13–22 (2014). 24878325 10.1016/j.vetmic.2014.04.019

[3] Collingwood, C., Kemmett, K., Williams, N. & Wigley, P. Is the Concept of Avian Pathogenic Escherichia coli as a Single Pathotype Fundamentally Flawed? *Front. Vet. Sci.* **1**, 5. 10.3389/fvets.2014.00005 (2014). 26664913 10.3389/fvets.2014.00005 PMC4668852

[4] Dziva, F. & Stevens, M. P. Colibacillosis in poultry: unravelling the molecular basis of virulence of avian pathogenic Escherichia coli in their natural hosts. *Avian Pathol.* **37**, 355–366. 10.1080/03079450802216652 (2008). 18622850 10.1080/03079450802216652

[5] Guabiraba, R. & Schouler, C. Avian colibacillosis: still many black holes. *FEMS Microbiol. Lett.* **362**, fnv118. 10.1093/femsle/fnv118 (2015). 26204893 10.1093/femsle/fnv118

[6] Shahcheraghi, F. et al. Identification and characterization of class 1 integrons among atypical enteropathogenic Escherichia coli isolated from children under 5 years of age. *Iran. J. Microbiol.* **6**, 156–162 (2014). 25870748 PMC4393491

[7] Johnson, J. R. & Russo, T. A. Extraintestinal pathogenic Escherichia coli: The other bad E coli. *J. Lab. Clin. Med.* **139**, 155–162. 10.1067/mlc.2002.121550 (2002). 11944026 10.1067/mlc.2002.121550

[8] Ovi, F., Zhang, L., Nabors, H., Jia, L. & Adhikari, P. A compilation of virulence-associated genes that are frequently reported in avian pathogenic Escherichia coli (APEC) compared to other E. coli. *J. Appl. Microbiol.* **134**, lxad014 (2023). 36754368 10.1093/jambio/lxad014

[9] Cella, E. et al. Joining forces against antibiotic resistance: The one health solution. *Pathogens* **12**, 1074 (2023). 37764882 10.3390/pathogens12091074 PMC10535744

[10] Sonola, V. S., Katakweba, A. S., Misinzo, G. & Matee, M. I. Occurrence of multi-drug-resistant Escherichia coli in chickens, humans, rodents and household soil in Karatu, Northern Tanzania. *Antibiotics* **10**, 1137 (2021). 34572718 10.3390/antibiotics10091137 PMC8469054

[11] Mohammed, R., Nader, S. M., Hamza, D. A. & Sabry, M. A. Public health concern of antimicrobial resistance and virulence determinants in E. coli isolates from oysters in Egypt. *Sci. Rep.* **14**, 26977 (2024). 39505944 10.1038/s41598-024-77519-y PMC11541584

[12] Kuan, N. L., Chen, Y. P., Shien, J. H. & Yeh, K. S. Characteristics of the extended-spectrum-β-lactamase-producing Escherichia coli isolated from diseased livestock and poultry in Taiwan. *Sci. Rep.* **14**, 29459. 10.1038/s41598-024-80943-9 (2024). 39604539 10.1038/s41598-024-80943-9 PMC11603147

[13] Agyare, C., Boamah, V. E., Zumbi, C. N. & Osei, F. B. in *Antimicrobial resistance-A global threat*IntechOpen, (2018).

[14] Schwarz, S., Kehrenberg, C. & Walsh, T. Use of antimicrobial agents in veterinary medicine and food animal production. *Int. J. Antimicrob. Agents*. **17**, 431–437 (2001). 11397611 10.1016/s0924-8579(01)00297-7

[15] Johnson, T. J., Wannemuehler, Y. M. & Nolan, L. K. Evolution of the < i>iss Gene in < i>Escherichia coli. *Appl. Environ. Microbiol.* **74**, 2360–2369. 10.1128/AEM.02634-07 (2008). 18281426 10.1128/AEM.02634-07 PMC2293169

[16] Quinn, P. J. et al. *Veterinary microbiology and microbial disease* (Wiley, 2011).

[17] Algammal, A. M. et al. Virulence-determinants and antibiotic-resistance genes of MDR-E. coli isolated from secondary infections following FMD-outbreak in cattle. *Sci. Rep.* **10**, 19779. 10.1038/s41598-020-75914-9 (2020). 33188216 10.1038/s41598-020-75914-9 PMC7666185

[18] Wayne, P. CLSI performance standards for antimicrobial susceptibility testing. *CLSI supplements M*. **100**, 20–30 (2020).

[19] Magiorakos, A. P. et al. Multidrug-resistant, extensively drug-resistant and pandrug-resistant bacteria: an international expert proposal for interim standard definitions for acquired resistance. *Clin. Microbiol. Infect.* **18**, 268–281 (2012). 21793988 10.1111/j.1469-0691.2011.03570.x

[20] Christopher, A. F., Hora, S. & Ali, Z. Investigation of plasmid profile, antibiotic susceptibility pattern multiple antibiotic resistance index calculation of Escherichia coli isolates obtained from different human clinical specimens at tertiary care hospital in Bareilly-India. *Annals Trop. Med. & Public Health* **6**, 285-289 (2013).

[21] Clermont, O., Christenson, J. K., Denamur, E. & Gordon, D. M. The C lermont E scherichia coli phylo-typing method revisited: improvement of specificity and detection of new phylo‐groups. *Environ. Microbiol. Rep.* **5**, 58–65 (2013). 23757131 10.1111/1758-2229.12019

[22] Hu, Q. et al. Development of multiplex PCR assay for rapid detection of Riemerella anatipestifer, Escherichia coli, and Salmonella enterica simultaneously from ducks. *J. Microbiol. Methods*. **87**, 64–69. 10.1016/J.MIMET.2011.07.007 (2011). 21791228 10.1016/j.mimet.2011.07.007

[23] Ibekwe, A. M., Murinda, S. E. & Graves, A. K. Genetic diversity and antimicrobial resistance of Escherichia coli from human and animal sources uncovers multiple resistances from human sources. *PloS one*. **6**, e20819 (2011). 21687635 10.1371/journal.pone.0020819 PMC3110821

[24] Randall, L., Cooles, S., Osborn, M., Piddock, L. & Woodward, M. J. Antibiotic resistance genes, integrons and multiple antibiotic resistance in thirty-five serotypes of Salmonella enterica isolated from humans and animals in the UK. *J. Antimicrob. Chemother.* **53**, 208–216 (2004). 14729766 10.1093/jac/dkh070

[25] Robicsek, A., Jacoby, G. A. & Hooper, D. C. The worldwide emergence of plasmid-mediated quinolone resistance. *Lancet. Infect. Dis*. **6**, 629–640 (2006). 17008172 10.1016/S1473-3099(06)70599-0

[26] Archambault, M. et al. Molecular characterization and occurrence of extended-spectrum β-lactamase resistance genes among Salmonella enterica serovar Corvallis from Thailand, Bulgaria, and Denmark. *Microb. Drug Resist.* **12**, 192–198 (2006). 17002546 10.1089/mdr.2006.12.192

[27] Colom, K. et al. Simple and reliable multiplex PCR assay for detection of bla TEM, bla SHV and bla OXA–1 genes in Enterobacteriaceae. *FEMS Microbiol. Lett.* **223**, 147–151 (2003). 12829279 10.1016/S0378-1097(03)00306-9

[28] Shibata, N. et al. PCR typing of genetic determinants for metallo-β-lactamases and integrases carried by gram-negative bacteria isolated in Japan, with focus on the class 3 integron. *J. Clin. Microbiol.* **41**, 5407–5413 (2003). 14662918 10.1128/JCM.41.12.5407-5413.2003 PMC309014

[29] Ewers, C. et al. Avian pathogenic, uropathogenic, and newborn meningitis-causing Escherichia coli: how closely related are they? *Int. J. Med. Microbiol.* **297**, 163–176 (2007). 17374506 10.1016/j.ijmm.2007.01.003

[30] Bisi-Johnson, M. A., Obi, C. L., Vasaikar, S. D., Baba, K. A. & Hattori, T. Molecular basis of virulence in clinical isolates of Escherichia coli and Salmonella species from a tertiary hospital in the Eastern Cape, South Africa. *Gut pathogens*. **3**, 9 (2011). 21663681 10.1186/1757-4749-3-9 PMC3125331

[31] Yaguchi, K. et al. Virulence factors of avian pathogenic Escherichia coli strains isolated from chickens with colisepticemia in Japan. *Avian Dis.* **51**, 656–662 (2007). 17992922 10.1637/0005-2086(2007)51[656:VFOAPE]2.0.CO;2

[32] Amer, M. M., Mekky, H. M., Amer, A. M. & Fedawy, H. S. Antimicrobial resistance genes in pathogenic Escherichia coli isolated from diseased broiler chickens in Egypt and their relationship with the phenotypic resistance characteristics. *Veterinary World*. **11**, 1082 (2018). 30250367 10.14202/vetworld.2018.1082-1088 PMC6141278

[33] Ibrahim, R. A. et al. Identification of Escherichia coli from broiler chickens in Jordan, their antimicrobial resistance, gene characterization and the associated risk factors. *BMC Vet. Res.* **15**, 159. 10.1186/s12917-019-1901-1 (2019). 31118039 10.1186/s12917-019-1901-1 PMC6530146

[34] Berkhoff, H. A. & Vinal, A. C. Congo Red Medium to Distinguish between Invasive and Non-Invasive Escherichia coli Pathogenic for Poultry. *Avian Dis.* **30**, 117–121. 10.2307/1590621 (1986). 3524540

[35] El-Tarabili, R. M., Hanafy, A. S. T., El Feky, T. M. & Virulence Resistance Profile, Antimicrobial Resistance Genes of ESBLs, XDR Escherichia coli Isolated from Ducks. *J. Adv. Veterinary Res.* **13**, 425–430 (2023).

[36] Kocúreková, T., Karahutová, L. & Bujňáková, D. Antimicrobial susceptibility and detection of virulence-associated genes in Escherichia coli strains isolated from commercial broilers. *Antibiotics* **10**, 1303 (2021). 34827241 10.3390/antibiotics10111303 PMC8614860

[37] Wang, M. C. et al. Characterization of uropathogenic Escherichia coli phylogroups associated with antimicrobial resistance, virulence factor distribution, and virulence-related phenotypes. *Infect. Genet. Evol.* **114**, 105493 (2023). 37634856 10.1016/j.meegid.2023.105493

[38] Luo, S. et al. Resistance and virulence gene analysis and molecular typing of Escherichia coli from duck farms in Zhanjiang, China. *Front. Cell. Infect. Microbiol.* **13**, 1202013 (2023). 37396302 10.3389/fcimb.2023.1202013 PMC10308044

[39] Sorour, H. K., Samir, A. & Orady, R. Anti-bacterial resistance of commensal escherichia coli strains of meconium origin in apparently healthy chicks. *Assiut Veterinary Med. J.* **70**, 238–249. 10.21608/avmj.2023.242845.1198 (2024).

[40] Ahmed, S. et al. Whole-genome characterization and global phylogenetic comparison of cefotaxime-resistant Escherichia coli isolated from broiler chickens. *J. Microbiol.* **63**, e2412009 (2025). 40313150 10.71150/jm.2412009 PMC13577006

[41] Aberkane, C., Messaï, A., Messaï, C. R. & Boussaada, T. Antimicrobial resistance pattern of avian pathogenic Escherichia coli with detection of extended-spectrum β-lactamase-producing isolates in broilers in east Algeria. *Veterinary world*. **16**, 449 (2023). 37041836 10.14202/vetworld.2023.449-454 PMC10082731

[42] Awad, A. M. et al. Incidence, pathotyping, and antibiotic susceptibility of avian pathogenic Escherichia coli among diseased broiler chicks. *Pathogens* **9**, 114 (2020). 32059459 10.3390/pathogens9020114 PMC7168244

[43] Abdel-Rahman, M. A. et al. Distribution pattern of antibiotic resistance genes in Escherichia coli isolated from colibacillosis cases in broiler farms of Egypt. *Veterinary world*. **16**, 1 (2023). 36855348 10.14202/vetworld.2023.1-11 PMC9967716

[44] Halfaoui, Z., Menoueri, N. M. & Bendali, L. M. Serogrouping and antibiotic resistance of Escherichia coli isolated from broiler chicken with colibacillosis in center of Algeria. *Veterinary world* 10, 830 (2017). 10.14202/vetworld.2017.830-835 PMC5553156 28831231

[45] Irenge, L. M. et al. Whole-genome sequences of multidrug-resistant Escherichia coli in South-Kivu Province, Democratic Republic of Congo: characterization of phylogenomic changes, virulence and resistance genes. *BMC Infect. Dis.* **19**, 137. 10.1186/s12879-019-3763-3 (2019). 30744567 10.1186/s12879-019-3763-3 PMC6371417

[46] Falgenhauer, L. et al. Detection and Characterization of ESBL-Producing Escherichia coli From Humans and Poultry in Ghana. *Front. Microbiol.* 9–2018. 10.3389/fmicb.2018.03358 (2019). 10.3389/fmicb.2018.03358 PMC6340976 30697208

[47] Krumperman, P. H. Multiple antibiotic resistance indexing of Escherichia coli to identify high-risk sources of fecal contamination of foods. *Appl. Environ. Microbiol.* **46**, 165–170. 10.1128/aem.46.1.165-170.1983 (1983). 6351743 10.1128/aem.46.1.165-170.1983 PMC239283

[48] Kong, R. Y. C., So, C. L., Law, W. F. & Wu, R. S. S. A Sensitive and Versatile Multiplex PCR System for the Rapid Detection of Enterotoxigenic (ETEC), Enterohaemorrhagic (EHEC) and Enteropathogenic (EPEC) Strains of Escherichia coli. *Mar. Pollut. Bull.* **38**, 1207–1215. 10.1016/S0025-326X(99)00164-2 (1999).

[49] Karunakaran, T. & Gunasekaran, P. Cloning and expression inEscherichia coli of an alkaline phosphatase (phoA) gene fromZymomonas mobilis. *Curr. Microbiol.* **25**, 291–295. 10.1007/BF01575864 (1992). 1369200 10.1007/BF01575864

[50] Rathi, A., Thong, K. L. & Chong, V. Isolation, detection and genomic differentiation of Escherichia coli from aquatic environments in Kelantan, Malaysia. *Malaysian J. Sci.* **29**, 1–10 (2009).

[51] Thong, K. L., Lai, M., Teh, C. S. J. & Chua, K. H. Simultaneous detection of methicillin-resistant Staphylococcus aureus, Acinetobacter baumannii, Escherichia coli, Klebsiella pneumoniae and Pseudomonas aeruginosa by multiplex PCR. *Trop Biomed.* **28**, 21-31 (2011). 21602765

[52] Saeed, E. et al. Prevalence, antibiotic sensitivity profile, and phylogenetic analysis of Escherichia coli isolated from raw dromedary camel milk in Matrouh Governorate, Egypt. *J. Adv. Veterinary Anim. Res.* **9**, 138 (2022). 10.5455/javar.2022.i578 PMC8985882 35445123

[53] Hu, Q. et al. Development of multiplex PCR assay for rapid detection of Riemerella anatipestifer, Escherichia coli, and Salmonella enterica simultaneously from ducks. *J. Microbiol. Methods*. **87**, 64–69 (2011). 21791228 10.1016/j.mimet.2011.07.007

[54] Alnahass, R., Khaliel, S., Ellakany, H. & Ibrahim, M. S. Comparison between Bacteriological Isolation and Molecular Detection of E. coli from Chickens Suffering from Colibacillosis and/or Diarrhea. *Alexandria J. Veterinary Sciences.* **49**, 141-148 (2016).

[55] Afayibo, D. J. A. et al. Isolation, molecular characterization, and antibiotic resistance of avian pathogenic Escherichia coli in Eastern China. *Veterinary Sci.* **9**, 319 (2022). 10.3390/vetsci9070319 PMC9324180 35878336

[56] Kiliç, A., Ertaş, H. B., Muz, A., Özbey, G. & Kalender, H. Detection of the eaeA gene in Escherichia coli from chickens by polymerase chain reaction. *Turkish J. Veterinary Anim. Sci.* **31**, 215–218 (2007).

[57] Moawad, A. A. et al. Antimicrobial resistance in Enterobacteriaceae from healthy broilers in Egypt: emergence of colistin-resistant and extended-spectrum β-lactamase-producing Escherichia coli. *Gut Pathogens*. **10**, 39. 10.1186/s13099-018-0266-5 (2018). 30250514 10.1186/s13099-018-0266-5 PMC6148799

[58] Ahmed, A. M., Shimamoto, T. & Shimamoto, T. Molecular characterization of multidrug-resistant avian pathogenic Escherichia coli isolated from septicemic broilers. *Int. J. Med. Microbiol.* **303**, 475–483 (2013). 23891276 10.1016/j.ijmm.2013.06.009

[59] Carattoli, A. Plasmids and the spread of resistance. *Int. J. Med. Microbiol.* **303**, 298–304 (2013). 23499304 10.1016/j.ijmm.2013.02.001

[60] Ahmed, M. F. E. et al. Occurrence of extended-spectrum beta-lactamase-producing Enterobacteriaceae, microbial loads, and endotoxin levels in dust from laying hen houses in Egypt. *BMC Vet. Res.* **16**, 301. 10.1186/s12917-020-02510-4 (2020). 32838780 10.1186/s12917-020-02510-4 PMC7446189

[61] Valverde, A. et al. Dramatic increase in prevalence of fecal carriage of extended-spectrum β-lactamase-producing Enterobacteriaceae during nonoutbreak situations in Spain. *J. Clin. Microbiol.* **42**, 4769–4775 (2004). 15472339 10.1128/JCM.42.10.4769-4775.2004 PMC522353

[62] Ahmed, M. O. et al. Analysis of Risk Factors Associated with Antibiotic-Resistant Escherichia coli. *Microb. Drug Resist.* **18**, 161–168. 10.1089/mdr.2011.0213 (2012). 22229818 10.1089/mdr.2011.0213

[63] Agusi, E. R. et al. Prevalence of multidrug-resistant Escherichia coli isolates and virulence gene expression in poultry farms in Jos, Nigeria. *Front. Microbiol.* 15–2024. 10.3389/fmicb.2024.1298582 (2024). 10.3389/fmicb.2024.1298582 PMC11199394 38933030

[64] Peirano, G. & Pitout, J. D. D. Extended-Spectrum β-Lactamase-Producing Enterobacteriaceae: Update on Molecular Epidemiology and Treatment Options. *Drugs* **79**, 1529–1541. 10.1007/s40265-019-01180-3 (2019). 31407238 10.1007/s40265-019-01180-3

[65] Antunes, P., Machado, J., Sousa, J. C. & Peixe, L. s. Dissemination of sulfonamide resistance genes (sul1, sul2, and sul3) in Portuguese Salmonella enterica strains and relation with integrons. *Antimicrobial agents and chemotherapy* 49, 836–839 (2005). 10.1128/AAC.49.2.836-839.2005 PMC547296 15673783

[66] El-Tarabili, R. M. et al. Prevalence, antibiotic profile, virulence determinants, ESBLs, and non-β-lactam encoding genes of MDR Proteus spp. isolated from infected dogs. *Front. Genet.* **13**, 952689 (2022). 36276974 10.3389/fgene.2022.952689 PMC9583872

