# Supplementary material for: Emergence of multidrug-resistant and virulent Escherichia coli with APEC‑associated traits in broiler chickens from Ismailia, Egypt
Source: Sci Rep. 2026 Apr 11;16:12067. doi: 10.1038/s41598-026-45788-4 (PMC13070038; doi:10.1038/s41598-026-45788-4)
Supplement: Supplementary file 1 — Supplementary Material 1 [file 41598_2026_45788_MOESM1_ESM.docx]

| **Antimicrobial class/ subclass** | **Antimicrobial agents** | **No. of *E. coli* isolates (%)** | | |
| --- | --- | --- | --- | --- |
|  |  | **Sensitive (S)** | **Intermediate (I)** | **Resistant (R)** |
| **Penicillins** | **Ampicillin** | 0 (0) | 0 (0) | 57 (100) |
| **β-lactams combination agents** | **Amoxicillin-clavulanic acid** | 3 (5.3) | 0 (0) | 54 (94.7) |
| **Cephalosporins ӀӀӀ** | **Ceftriaxone** | 4 (7) | 0 (0) | 53 (93) |
|  | **Cefuroxime** | 4 (7) | 0 (0) | 53 (93) |
| **Carbapenems** | **Imipenem** | 38 (66.7) | 3 (5.3) | 16 (28) |
| **Fluoroquinolones** | **Levofloxacin** | 29 (50.8) | 3 (5.3) | 25 (43.9) |
| **Tetracyclines** | **Tetracycline** | 0 (0) | 0 (0) | 57 (100) |
| **Sulfonamides** | **Trimethoprim-sulfamethoxazole** | 26 (43.9) | 1 (1.7) | 31 (54.4) |
| **Aminoglycosides** | **Gentamycin** | 6 (10.5) | 2 (3.5) | 49 (86) |

**Table 1S** : Results of antimicrobial susceptibility test of *E.coli* isolates


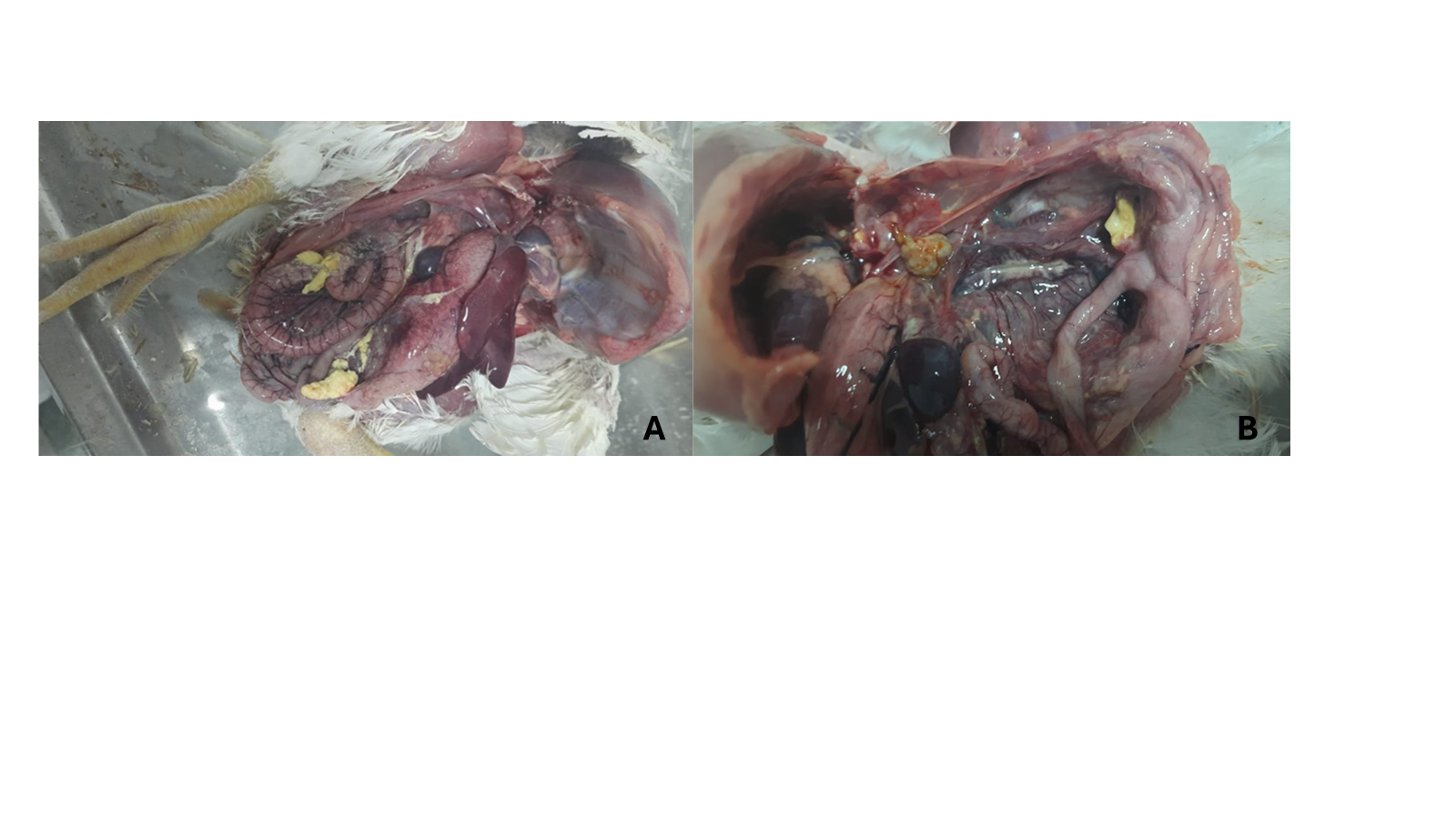


**Figure 1SA and B**: Yellow lumps are coated on the intestines and within the abdominal cavity, with accumulation of pus (fibrin) that has hardened. **A**: pale liver and looks "dusty" with a fibrinous layer on it, and **B**: congested intestine


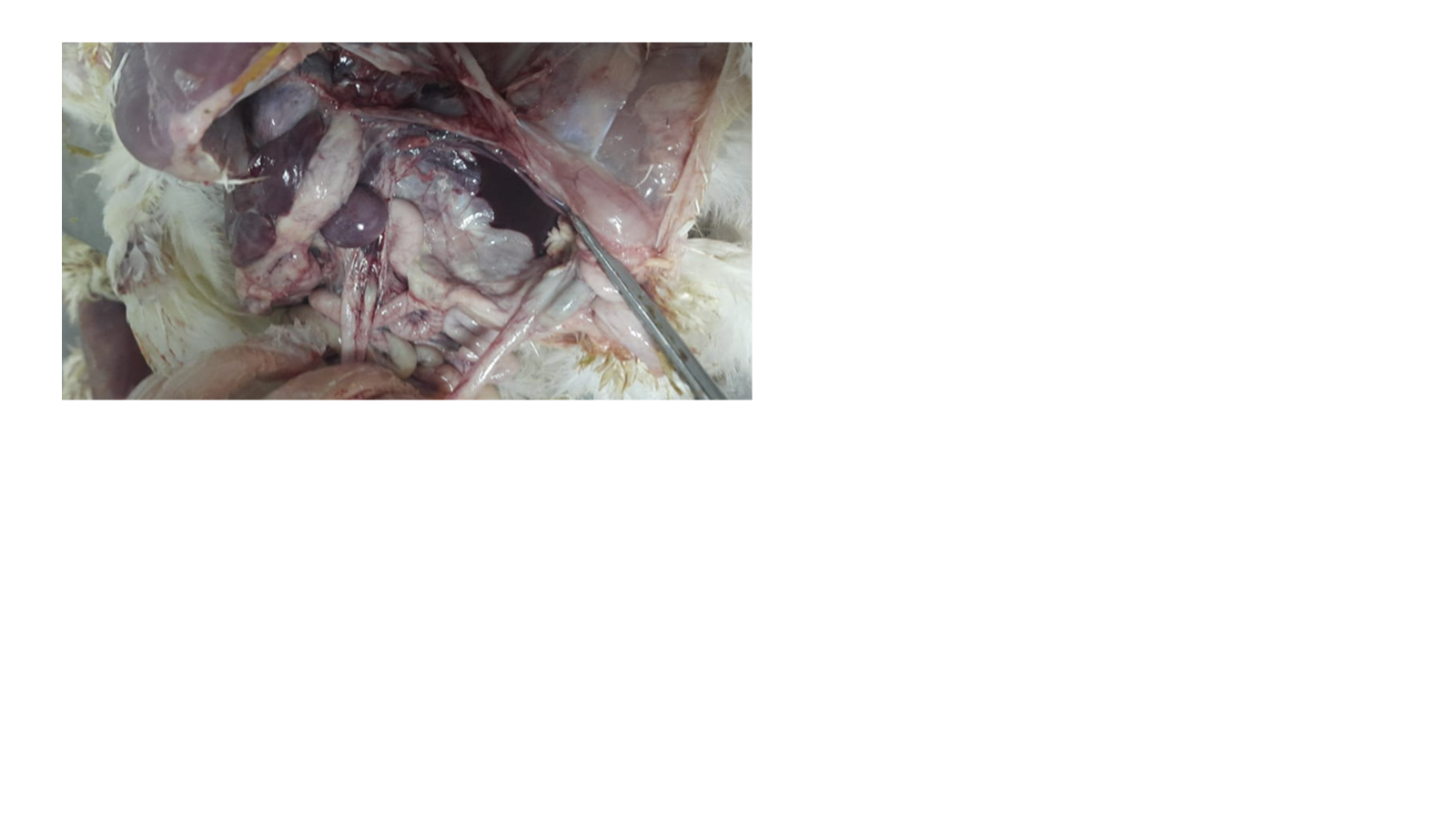


**Figure 2S:**The heart area is also cloudy and lacks the clearness characteristic of a healthy heart sac


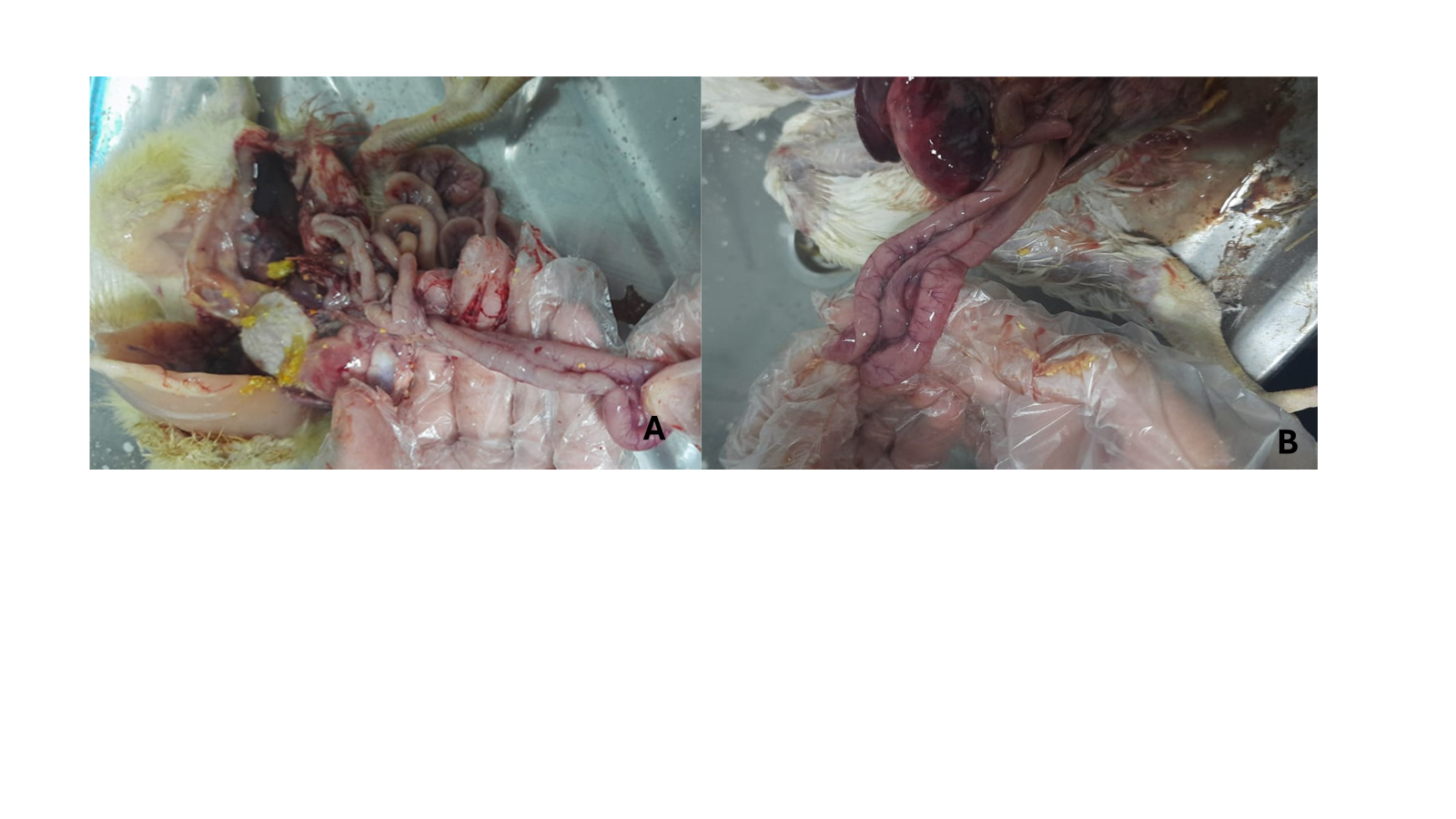


**Figure 3SA and B**: exhibited enteritis. **A**: The intestines look very red, irritated, and hemorrhagic. **B**: The blood vessels on the intestinal wall are hemorrhagic and prominent with hemorrhagic intestine.


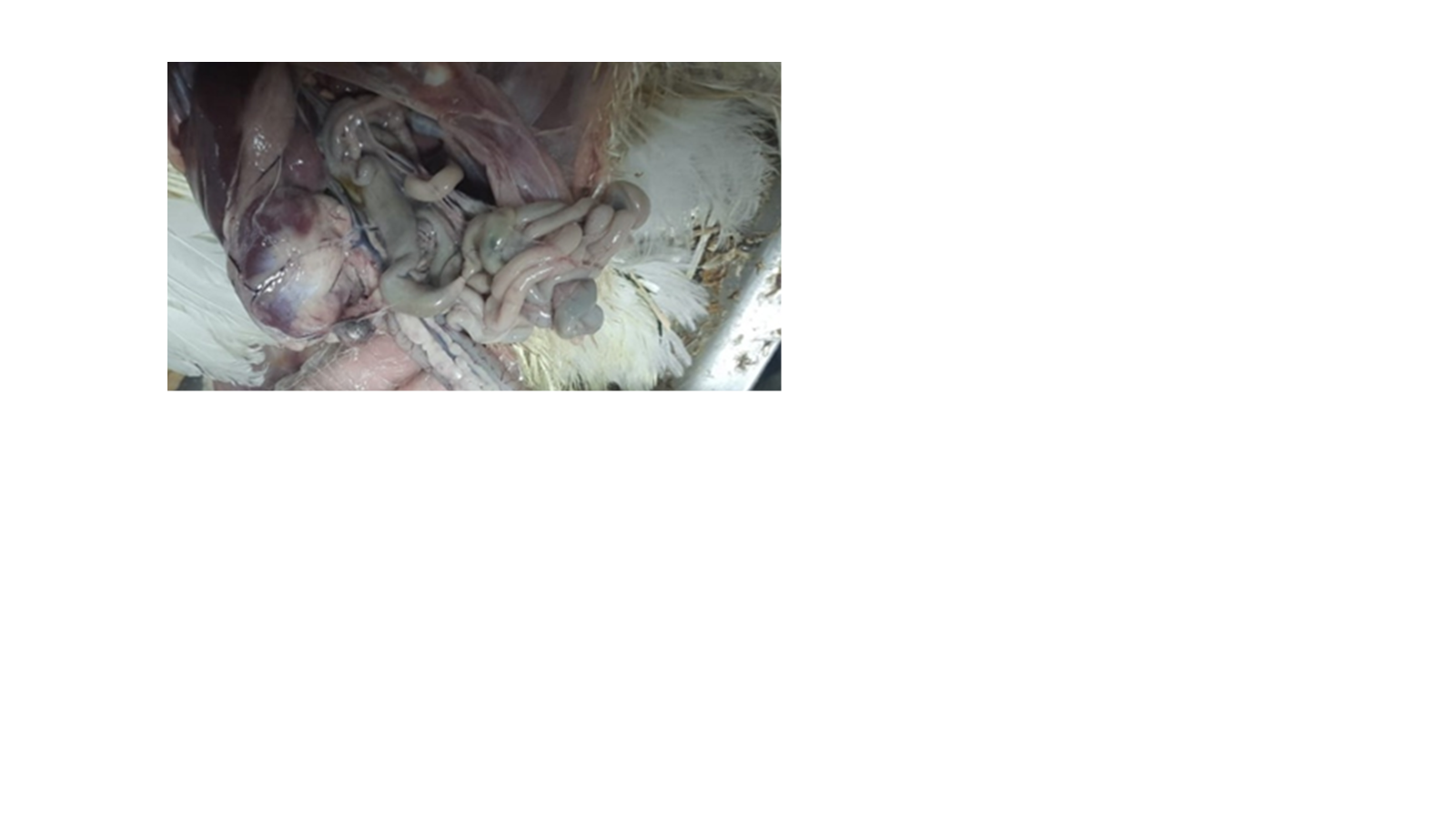


**Figure 4S:** The spleen looks very swollen, enlarged and hemorrhagic.


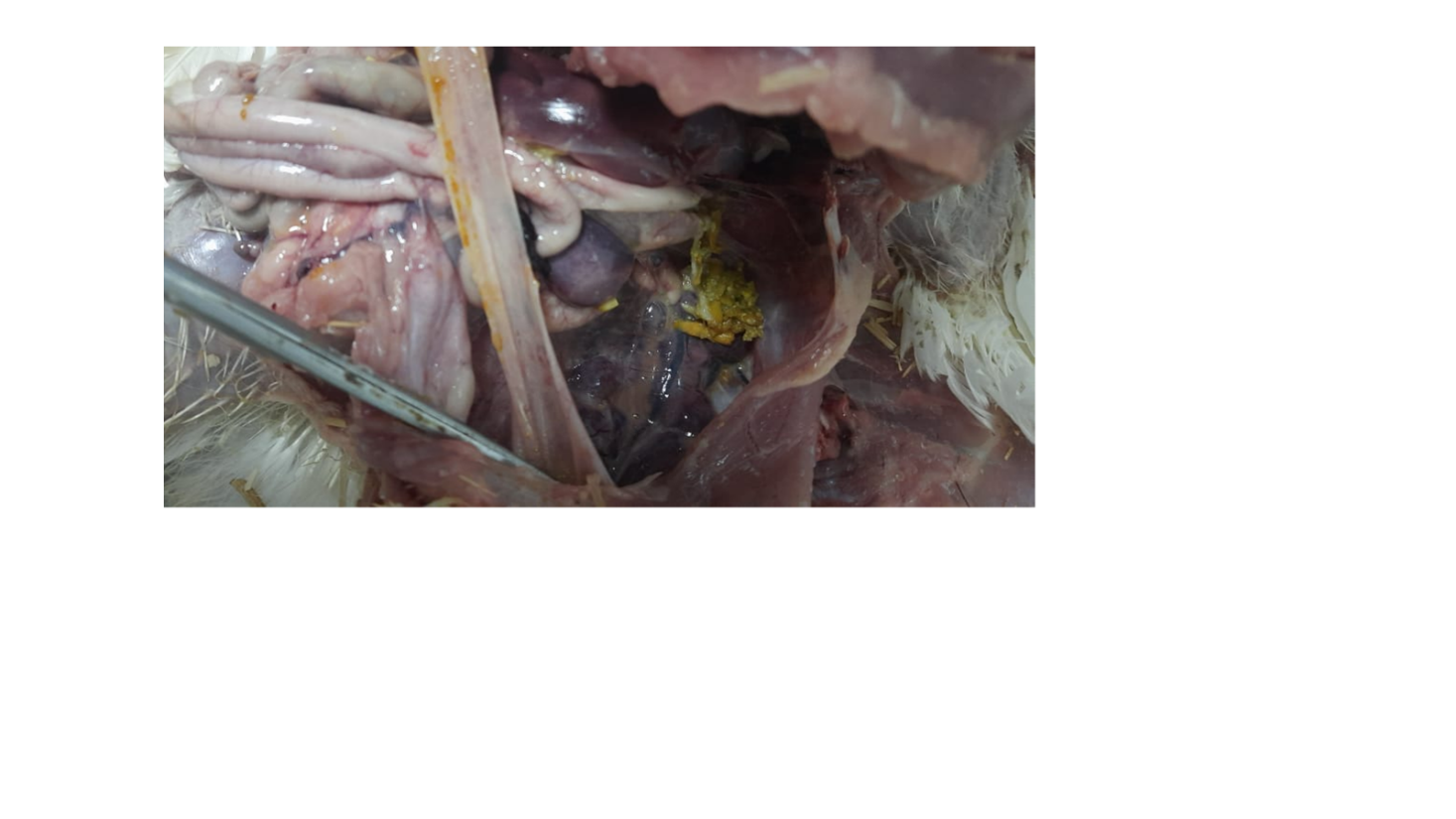


**Figure 5S:** Cloudy thickened air sacs with fibrin layer

**Supplementary Table S1:** Contingency table for SXT vs *sul*1

|  | **sul1 +** | **sul1 −** | **Total** |
| --- | --- | --- | --- |
| SXT resistant | 31 | 0 | 31 |
| SXT susceptible | 0 | 26 | 26 |
| Total | 31 | 26 | 57 |

**Supplementary Table S2**: Contingency table for levofloxacin vs *qnr*A

|  | **qnrA +** | **qnrA −** | **Total** |
| --- | --- | --- | --- |
| LEV resistant | 25 | 0 | 25 |
| LEV susceptible | 0 | 32 | 32 |
| Total | 25 | 32 | 57 |

**Supplementary Table S3:** Contingency table for gentamycin vs *aad*A1

|  | **aadA1 +** | **aadA1 −** | **Total** |
| --- | --- | --- | --- |
| Gentamycin resistant | 49 | 0 | 49 |
| Gentamycin susceptible | 0 | 8 | 8 |
| Total | 49 | 8 | 57 |

**Supplementary Table S4:** Contingency table for imipenem vs *bla*_IMP‑1_

|  | **blaIMP‑1 +** | **blaIMP‑1 −** | **Total** |
| --- | --- | --- | --- |
| Imipenem resistant | 16 | 0 | 16 |
| Imipenem susceptible | 0 | 41 | 41 |
| Total | 16 | 41 | 57 |

**Supplementary Table S5:** Contingency table for imipenem vs *bla*_VIM‑1_

|  | **blaVIM‑1 +** | **blaVIM‑1 −** | **Total** |
| --- | --- | --- | --- |
| Imipenem resistant | 16 | 0 | 16 |
| Imipenem susceptible | 3 | 38 | 41 |
| Total | 19 | 38 | 57 |
